# Intake of Ultra-processed Foods Is Associated with an Increased Risk of Crohn’s Disease: A Cross-sectional and Prospective Analysis of 187 154 Participants in the UK Biobank

**DOI:** 10.1093/ecco-jcc/jjac167

**Published:** 2022-10-28

**Authors:** Jie Chen, Judith Wellens, Rahul Kalla, Tian Fu, Minzi Deng, Han Zhang, Shuai Yuan, Xiaoyan Wang, Evropi Theodoratou, Xue Li, Jack Satsangi

**Affiliations:** School of Public Health and Second Affiliated Hospital, Zhejiang University School of Medicine, Hangzhou, Zhejiang, China; Department of Gastroenterology, Third Xiangya Hospital, Central South University, Changsha, China; Translational Gastro-Intestinal Unit, Nuffield Department of Medicine, John Radcliffe Hospital, Oxford, UK; KU Leuven Department of Chronic Diseases and Metabolism, Translational Research Center for Gastrointestinal Disorders, Leuven, Belgium; Medical Research Council Centre for Inflammation Research, Queens Medical Research Institute, University of Edinburgh, Edinburgh, UK; Department of Gastroenterology, Third Xiangya Hospital, Central South University, Changsha, China; Department of Gastroenterology, Third Xiangya Hospital, Central South University, Changsha, China; School of Public Health and Second Affiliated Hospital, Zhejiang University School of Medicine, Hangzhou, Zhejiang, China; Unit of Cardiovascular and Nutritional Epidemiology, Institute of Environmental Medicine, Karolinska Institutet, Stockholm, Sweden; Department of Gastroenterology, Third Xiangya Hospital, Central South University, Changsha, China; Centre for Global Health Research, Usher Institute, University of Edinburgh, Edinburgh, UK; Cancer Research UK Edinburgh Centre, MRC Institute of Genetics and Molecular Medicine, University of Edinburgh, Edinburgh, UK; School of Public Health and Second Affiliated Hospital, Zhejiang University School of Medicine, Hangzhou, Zhejiang, China; Translational Gastro-Intestinal Unit, Nuffield Department of Medicine, John Radcliffe Hospital, Oxford, UK

**Keywords:** Inflammatory bowel diseases, ultra-processed food, utrition

## Abstract

**Background and Aims:**

Ultra-processed food [UPF] consumption has been linked to globally increasing incidence and prevalence of chronic diseases, including inflammatory bowel diseases [IBD]. We aimed to investigate the association between UPF consumption and IBD incidence, prevalence, and IBD-relevant outcomes.

**Methods:**

We performed a cross-sectional and prospective cohort study in 187 854 individuals included in the national UK Biobank, using 24-h dietary recall questionnaires. Multivariable logistic regression and Cox proportional hazard regression were used to examine the association between UPFs and the prevalence and incidence risk of IBD, respectively.

**Results:**

A total of 185 849 participants with a mean age of 56.2 were included, with a mean follow-up of 9.84 years. During follow-up, 841 developed IBD (251 Crohn’s disease [CD], and 590 ulcerative colitis [UC]). UPF intake in IBD patients was significantly higher: CD: odds ratio [OR] 1.94 (95% confidence interval [CI]: 1.52, 2.49, *p* <0.001); UC: OR 1.39 [95% CI: 1.17, 1.65, *p* <0.001]. Compared with low consumption, higher UPF consumption was significantly associated with incident CD: hazard ration [HR] 2.00 [95% CI: 1.32, 3.03, *p* = 0.001], but not UC. We also found a significant association between UPF intake and need of IBD-related surgery: HR 4.06 [95% CI: 1.52, 10.86, *p* = 0.005].

**Conclusion:**

Higher intake of UPFs was associated with higher incidence of CD, but not UC. In individuals with a pre-existing diagnosis of IBD, consumption of UPFs was significantly higher compared with controls, and was associated with an increased need for IBD-related surgery. Further studies are needed to address the impact of UPF intake on disease pathogenesis and outcomes.

## Graphical abstract

**Graphical Abstract:**
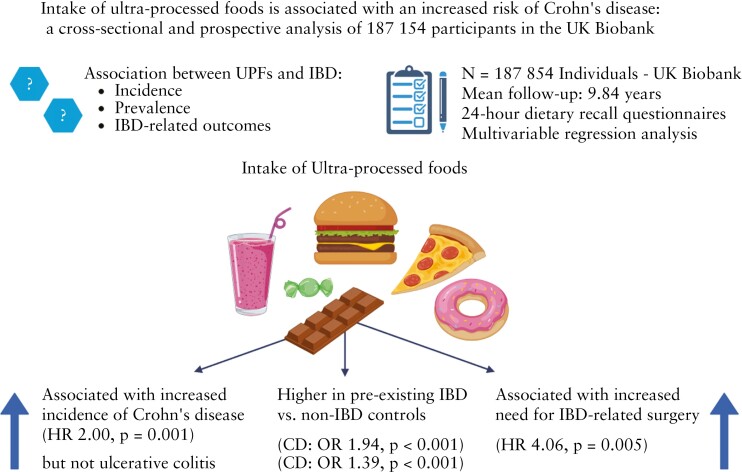

## 1. Introduction

Ultra-processed foods [UPFs] make up more than half of the total dietary energy consumed in many high-income countries.^1,2^ Although most foods are processed to some extent, UPFs are formulations of ingredients that result from a series of industrial processes, as defined by the NOVA classification system.^2,3^ UPFs have been recognised as energy-dense products, high in sugar, unhealthy fats, and salt, and low in dietary fibre, protein, vitamins, and minerals. Cross-sectional and longitudinal studies have shown that increases in the dietary proportion of UPFs result in deterioration of the nutritional quality of the overall diet and increased obesity, hypertension, coronary and cerebrovascular diseases, dyslipidaemia, metabolic syndrome, cancer, and gastrointestinal disorders.^2^ In addition, experimental studies indicate that UPFs can induce high glycaemic responses, have a low satiety potential, and create a pro-inflammatory gut environment.^4^

Inflammatory bowel diseases [IBD] are chronic inflammatory conditions of the gastrointestinal tract and comprised two main entities, namely Crohn’s disease [CD] and ulcerative colitis [UC]. Although historically these have been considered to be Western diseases, incidence and prevalence are increasing globally, particlarly in industrialised and industrialising regions of the world such as Asia, the Middle East and Latin America.^5,6^ In order to explain these increasing incidence rates, the role of diet has been closely examined. High dietary intake of total fats, polyunsaturated fatty acids [PUFAs], omega-6 fatty acids, meat, and sugar-sweetened beverages have been associated with an increased risk of CD and UC in observational studies.^7,8^ Other than macronutrients, the non-nutritional or ‘organoleptic characteristic’ components in our diet such as emulsifiers and colourants, have recently been implicated as playing a role in driving inflammation and metabolic derangement in a number of animal and *in vitro* studies.^9–11^

A number of recent studies have assessed the association between UPFs and IBD, although to date the findings have been inconsistent.^12–14^ The French NutriNet-Santé cohort did not find any significant association between UPF intake and IBD incidence.^13^ Most recently, however, two larger studies have been completed. The global PURE cohort reported a positive association between UPF intake and risk for IBD, whereas in the American Nurses’ Health Study cohort, the authors reported only on an association of UPF intake with CD.^12,14^ These findings require to be further validated. In this study we aimed to investigate the association between UPF consumption and IBD incidence, prevalence, and IBD-relevant outcomes, in the UK Biobank.

## 2. Materials and Methods

### 2.1. Study design and participants

**Figure 1.**
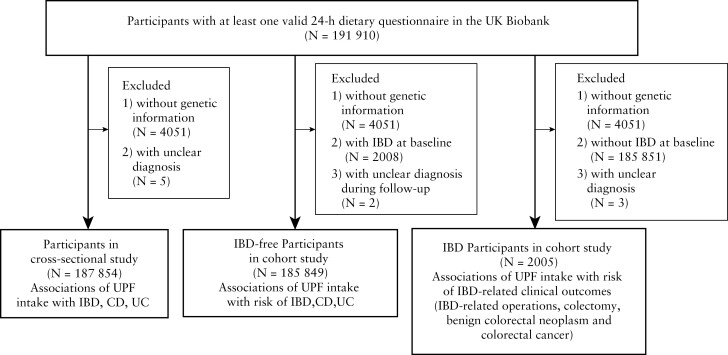
Flowchart of the selection of eligible study population. IBD, inflammatory bowel diseases; CD, Crohn’s disease; UC, ulcerative colitis.

The current study was conducted in the UK Biobank, which is a large cohort study incorporating over 500 000 participants, aged 40–69 years, from 2006 to 2010 in the UK. Further details of the study have been described elsewhere.^15^ In this study, 191 910 participants had at least one valid 24-h dietary recall questionnaire with credible energy records [>0 and <18 MJ for females, >0 and <20 MJ for males] and were included in the analysis [Figure 1].^16^ Three separate sub-studies were constructed: a cross-sectional study with IBD patients at baseline [according to hospital diagnosis or general practice reports, thereby including all prevalent cases] and participants without IBD, a prospective cohort with participants without IBD at baseline to investigate IBD incidence, and another prospective cohort with IBD patients only, to investigate the influence of UPF intake on relevant disease outcomes such as colorectal neoplasia and need for IBD-related surgery. Participants were excluded when no dietary information was available, when the type of IBD diagnosis was left unspecified, or genetic information was unavailable.

### 2.2. Exposure and outcome measurements

UPFs were defined according to the NOVA classification.^1,2^ The Oxford WebQ questionnaire used by UK Biobank contained 206 food items and 32 alcohol and beverage items to assess dietary consumption over the past 24 h. The 24-h WebQ questionnaire was previously validated with good agreement with the food frequency questionnaire of the UK Biobank and the mean intake of multiple measurements further reduces bias.^17,18^ Participants were asked to select how many portions they consumed for each item, with instructions specifying what one portion size represented, such as one sausage, one rasher of bacon, one slice of ham, or one ‘serving’ for some specific foods. When multiple rounds of dietary recalls were available for the same participant, the mean value was taken into account. The food intake weight in grams for each item was calculated by multiplying amounts of portion size by standard portion sizes in grams; then daily intakes of energy and nutrients were estimated by multiplying the food weight consumed by its nutrient composition. Portion size and nutrient and energy compositions for each food item used for UPF estimation were calculated according to the UK McCance and Widdowson’s *The Composition of Foods*, 6th edition [2002] and its supplements as defined by the NOVA classification.^19,20^ Food items included for the estimation of UPF intake are presented in Supplementary Table 1. Intake of each single UPF was calculated as the mean intake of each valid 24-h dietary recall questionnaire, and UPF consumption was calculated as the sum of all these dietary elements. Consumption was further divided into number of UPF servings, energy intake from UPFs, and proportion of energy percentage from UPFs.

Diagnostic information was obtained from both primary care and hospital inpatient records containing data on admissions, diagnoses, and operation procedures. The primary outcomes include the prevalence and incidence of CD and UC, IBD-related surgical operations [colectomy and other operations], and IBD-related complications [benign colorectal neoplasms, colorectal cancer]. Prevalent and incident CD and UC cases were ascertained by a primary or secondary diagnosis defined by the corresponding International Classification of Diseases codes [ICD-9: 555, 556; ICD-10: K50, K51]. Participants without IBD at baseline were followed up from baseline [2006–10] until the date of first diagnosis of CD or UC, date of death, date of loss to follow-up, or the last date of follow-up, whichever came first. IBD patients were followed up for their disease outcomes, including surgical operations [colectomy and other operations] and long-term clinical outcomes [ie, benign colorectal neoplasms and colorectal cancer]. CD location was categorised as ileal [L1], colonic [L2], ileocolonic [L3], or location not defined [LX], and UC extent was categorised as proctitis [E1], left-sided [E2], extensive [E3], or undefined [EX] UC, for subgroup analyses of the association with disease locations.

### 2.3. Covariate assessment

Information on covariates, including age, sex, ethnicity, education attainment, and Townsend Deprivation Index [TDI], was collected in the baseline questionnaire. Polygenic risk scores [PRS] were constructed to proxy the genetic propensity to CD and UC separately for each participant, by summing the number of risk-increasing alleles for genetic variants associated with CD or UC and weighted by their effect sizes, respectively, as reported by previous genome-wide association study of IBD.^21^ Other measurements included smoking status, alcohol, physical activity, body mass index [BMI], C-reactive protein [CRP], urine sodium, dietary factors [nutrient intake, total energy intake], alternative healthy eating index [AHEI], comorbidities [Charlson Comorbidity Index [CCI]], family history of bowel cancer, IBD-related medication [glucocorticoid, immunosuppressants, 5-aminosalicylic acid, monoclonal] were also considered as covariates for adjustment.^22–24^ AHEI was constructed by five food items [red meat, processed meat, fruit, vegetables, and fat] according to Anderson *et al.*, with higher score representing a healthier diet.^25^ CCI was defined using the method developed by Quan *et al.* [based on ICD-10 and enhanced ICD-9-CM] with data from hospital inpatient records (Hospital Episode Statistics [HES] dataset).^26^

### 2.4. Statistical analyses

Multivariable logistic regression models were used to examine the association between the UPF intakes [measured as UPF servings, energy intake from UPFs, and proportion of energy percentage from UPFs] and the prevalent risk of CD, UC, and combined as IBD, for the cross-sectional study. Cox proportional hazard regression models were performed to examine the associations of UPF intakes and the incident risk of CD, UC, and combined as IBD in the first prospective cohort study, and to examine the influence of UPF intake on the risk of IBD-related surgical operations and clinical complications in the second prospective cohort study of IBD patients. The minimally adjusted model was adjusted for age, age squared, sex, and ethnicity, and the fully adjusted model was further adjusted for TDI, smoking status, alcohol intake, education level, physical activity, BMI, total energy, and polygenic risk scores. The fully adjusted model for the risk of IBD-related surgical operations and clinical complications was additionally adjusted for disease features, including disease location, duration, and behaviours, age at diagnosis, medication use, presence of systemic symptoms [fever and weight loss], and family history of bowel cancer [when the outcome of interest was colorectal neoplasia].

Further analyses were conducted for associations between UPF intake and IBD risk. Secondary analyses included association of IBD risk according to UPF subgroups, disease location, and subgroup analyses stratified by relevant covariates.^27^ Sensitivity analyses were performed with further adjustment for nutrient intake [intake of total fat, carbohydrate, and protein, total sugar and fibre, saturated fat, and polyunsaturated fat], AHEI, urine sodium, CRP, and CCI. Participants with implausible energy intake [men with <800 or >4200 kcal/day, or women with 600 or > 3500 kcal/day] were excluded,^28^ missing covariates were explored using multiple imputation, liquid UPF foods were excluded, and all data were analysed excluding all IBD diagnoses within 1 year after recruitment. Results are presented as odds ratios [OR] or hazard ratios [HR] with 95% confidence intervals [CI]. The Bonferroni correction was applied to correct for multiple testing, for which we explored three different measures of UPF intake and three primary outcomes of interest, resulting in a *p*-value <0.0056 as the significance threshold for the main analysis. All statistical analyses were performed using R 4.1.3.

## 2.5. Ethical statement

The UK Biobank received ethical approval from the North West-Haydock Research Ethics Committee [REC reference: 16/NW/0274]. All participants in this study provided informed consent when they were recruited.

## 3. Results

### 3.1. Individuals with a known pre-existing diagnosis of IBD consumed significantly more UPFs than participants without IBD

In the cross-sectional study, a total of 187 854 participants were included, of whom 680 had a diagnosis of CD and 1325 had a diagnosis of UC at baseline. Of all included participants, 102 890 were female [54.8%], the mean age was 56.2 (standard deviation [SD] = 7.9), 95.9% of participants was of White ethnicity, the mean BMI was 26.9 kg/m^2^ [SD = 4.6], and total energy intake was 8666.4 KJ [SD = 2453.5; Table 1].

**Table 1.** Characteristics and UPF intake of participants according to IBD status and subtype in the cross-sectional study

	Overall [*n* = 187 854]	Non-IBD [*n* = 185849]	CD [*n* = 680]	UC [*n* = 1325]
Sex [%]				
Female	102 890 [54.8]	101 864 [54.8]	370 [54.4]	656 [49.5]
Male	84 964 [45.2]	83 985 [45.2]	310 [45.6]	669 [50.5]
Age, mean [SD]	56.2 [7.9]	56.2 [7.9]	55.7 [8.0]	57.2 [7.8]
Ethnicity [%]				
White	180 238 [95.9]	178 299 [95.9]	658 [96.8]	1281 [96.7]
Other	7616 [4.1]	7550 [4.1]	22 [3.2]	44 [3.3]
TDI				
High deprivation	62 617 [33.3]	61 924 [33.3]	242 [35.6]	451 [34.0]
Low deprivation	62 636 [33.3]	61 988 [33.4]	232 [34.1]	416 [31.4]
Moderate deprivation	62 601 [33.3]	61 937 [33.3]	206 [30.3]	458 [34.6]
Education level [%]				
College and above	79 738 [42.4]	79 013 [42.5]	234 [34.4]	491 [37.1]
High school and below	108 116 [57.6]	106 836 [57.5]	446 [65.6]	834 [62.9]
Smoking [%]				
Current	14 416 [7.7]	14 291 [7.7]	62 [9.1]	63 [4.8]
Never	106 716 [56.8]	105 753 [56.9]	314 [46.2]	649 [49.0]
Previous	66 722 [35.5]	65 805 [35.4]	304 [44.7]	613 [46.3]
Alcohol drinking [%]				
Current	175 886 [93.6]	174 051 [93.7]	613 [90.1]	1222 [92.2]
Never	6164 [3.3]	6087 [3.3]	28 [4.1]	49 [3.7]
Previous	5804 [3.1]	5711 [3.1]	39 [5.7]	54 [4.1]
BMI, mean [SD]	26.9 [4.6]	26.9 [4.6]	26.3 [4.4]	26.9 [4.6]
Physical activity [%]				
High	62954 [33.5]	62359 [33.6]	197 [29.0]	398 [30.0]
Low	29008 [15.4]	28635 [15.4]	143 [21.0]	230 [17.4]
Middle	67404 [35.9]	66706 [35.9]	224 [32.9]	474 [35.8]
NA	28488 [15.2]	28149 [15.1]	116 [17.1]	223 [16.8]
Total energy intake K, mean [SD]	8666.4 [2453.5]	8663.2 [2452.4]	9082.3 [2596.8]	8902.8 [2503.5]
Number of 24-h Q				
1	67426 [35.9]	66637 [35.9]	275 [40.4]	514 [38.8]
2	44428 [23.7]	43973 [23.7]	154 [22.6]	301 [22.7]
3	40729 [21.7]	40312 [21.7]	134 [19.7]	283 [21.4]
4	29634 [15.8]	29345 [15.8]	101 [14.9]	188 [14.2]
5	5637 [3.0]	5582 [3.0]	16 [2.4]	39 [2.9]
Serving, mean [SD]	8.5 [4.5]	8.5 [4.5]	9.8 [5.1]	9.0 [4.9]
Serving [%]				
Q1	36333 [19.3]	35994 [19.4]	93 [13.7]	246 [18.6]
Q2	38661 [20.6]	38310 [20.6]	105 [15.4]	246 [18.6]
Q3	37714 [20.1]	37373 [20.1]	116 [17.1]	225 [17.0]
Q4	35779 [19.0]	35345 [19.0]	164 [24.1]	270 [20.4]
Q5	39367 [21.0]	38827 [20.9]	202 [29.7]	338 [25.5]
Energy KJ, mean [SD]	3635.3 [1817.4]	3631.9 [1816.1]	4131.1 [2006.8]	3861.4 [1853.5]
Energy KJ [%]				
Q1	37571 [20.0]	37240 [20.0]	101 [14.9]	230 [17.4]
Q2	37571 [20.0]	37247 [20.0]	101 [14.9]	223 [16.8]
Q3	37570 [20.0]	37194 [20.0]	111 [16.3]	265 [20.0]
Q4	37571 [20.0]	37120 [20.0]	173 [25.4]	278 [21.0]
Q5	37571 [20.0]	37048 [19.9]	194 [28.5]	329 [24.8]
Energy proportion [%], mean [SD]	41.0 [15.0]	41.0 [15.0]	45.0 [16.0]	43 [16.0]
Energy proportion [%]				
Q1	37571 [20.0]	37224 [20.0]	97 [14.3]	250 [18.9]
Q2	37571 [20.0]	37221 [20.0]	115 [16.9]	235 [17.7]
Q3	37570 [20.0]	37220 [20.0]	117 [17.2]	233 [17.6]
Q4	37571 [20.0]	37128 [20.0]	158 [23.2]	285 [21.5]
Q5	37571 [20.0]	37056 [19.9]	193 [28.4]	322 [24.3]

Energy KJ [%] in indicated participants’ number [percentage] of each quintile [Q1, Q2, Q3, Q4, Q5] of energy intake from UPFs; Q1 and Q5 were the lowest and highest quintile of the studied population, based on energy of UPF consumption, respectively. Energy proportion [%] indicated participants’ number [percentage] of each quintile [Q1, Q2, Q3, Q4, Q5] of energy percentage from UPFs.

UPF, ultra-processed food; BMI, body mass index; IBD, inflammatory bowel diseases; SD, standard deviation; TDI, Townsend Deprivation Index; CD, Crohn’s disease; UC, ulcerative colitis; NA, not available.

Compared with non-IBD participants, there was a significant association between UPF intake [for number of servings, energy intake from UPF,s and percentage energy intake from UPFs] and prevalent cases of IBD in the fully adjusted model [adjusted for age, age squared, sex, and ethnicity, TDI, smoking status, alcohol intake, education level, physical activity, BMI, total energy, and polygenic risk scores] [Supplementary Table 2]. For UPFs as energy intake, compared with the lowest quintile of overall participants, we found an OR of 1.56 [95% CI: 1.35, 1.79, *p* <0.001], with an OR of 1.17 [95% CI: 1.12, 1.22, *p* <0.001] per SD and *p* <0.001 for the trend.

When analysing CD and UC separately, the results remained significant in the fully adjusted models, with an OR of 1.94 [95% CI: 1.52, 2.49, *p* <0.001] for UPF intake as energy intake and CD, and an OR of 1.39 [95% CI: 1.17, 1.65, *p* <0.001] for the same measurement for UC [Table 2].

**Table 2.** Association between UPF intake and CD, UC

	CD					UC				
	Cases	Minimally adjusted model OR 95% CI	*p*	Fully adjusted model OR 95% CI	*p*	Cases	Minimally adjusted model OR 95% CI	*p*	Fully adjusted model OR 95% CI	*p*
Serving										
Per SD		1.28 [1.20, 1.36]	**<0.001**	1.23 [1.14, 1.32]	**<0.001**		1.10 [1.04, 1.15]	**<0.001**	1.06 [1.00, 1.12]	**0.048**
Q1	93	ref		ref		246	ref		ref	
Q2	105	1.06 [0.80, 1.41]	0.670	1.03 [0.78, 1.37]	0.830	246	0.92 [0.77, 1.10]	0.373	0.90 [0.75, 1.08]	0.243
Q3	110	1.16 [0.88, 1.53]	0.300	1.10 [0.83, 1.46]	0.509	220	0.84 [0.70, 1.01]	0.067	0.81 [0.67, 0.98]	**0.027**
Q4	170	1.86 [1.45, 2.41]	**<0.001**	1.72 [1.32, 2.25]	**<0.001**	275	1.08 [0.91, 1.28]	0.399	1.01 [0.84, 1.21]	0.952
Q5	202	2.05 [1.60, 2.64]	**<0.001**	1.81 [1.38, 2.40]	**<0.001**	338	1.20 [1.01, 1.42]	**0.034**	1.08 [0.90, 1.31]	0.416
*p-*trend			**<0.001**		**<0.001**			**0.004**		0.160
Energy										
Per SD		1.28 [1.20, 1.37]	**<0.001**	1.27 [1.19, 1.36]	**<0.001**		1.12 [1.06, 1.18]	**<0.001**	1.11 [1.05, 1.17]	**<0.001**
Q1	101	ref		ref		229	ref		ref	
Q2	101	1.00 [0.76, 1.32]	0.999	1.01 [0.77, 1.33]	0.947	223	0.96 [0.80, 1.16]	0.677	0.97 [0.80, 1.16]	0.728
Q3	111	1.10 [0.84, 1.45]	0.478	1.11 [0.85, 1.46]	0.448	266	1.14 [0.95, 1.36]	0.150	1.14 [0.96, 1.37]	0.145
Q4	173	1.73 [1.35, 2.22]	**<0.001**	1.74 [1.36, 2.23]	**<0.001**	278	1.18 [0.99, 1.41]	0.063	1.19 [0.99, 1.42]	0.059
Q5	194	1.95 [1.53, 2.50]	**<0.001**	1.94 [1.52, 2.49]	**<0.001**	329	1.40 [1.18, 1.66]	**<0.001**	1.39 [1.17, 1.65]	**<0.001**
*p*-trend			**<0.001**		**<0.001**			**<0.001**		**<0.001**
Energy %										
Per SD		1.26 [1.17, 1.36]	**<0.001**	1.24 [1.15, 1.34]	**<0.001**		1.12 [1.06, 1.18]	**<0.001**	1.11 [1.05, 1.17]	**<0.001**
Q1	97	ref		ref		250	ref		ref	
Q2	115	1.18 [0.90, 1.55]	0.226	1.19 [0.91, 1.56]	0.209	235	0.93 [0.78, 1.11]	0.418	0.92 [0.77, 1.11]	0.391
Q3	116	1.19 [0.91, 1.56]	0.207	1.20 [0.92, 1.58]	0.187	232	0.91 [0.76, 1.09]	0.321	0.91 [0.76, 1.09]	0.306
Q4	159	1.63 [1.27, 2.11]	**<0.001**	1.62 [1.26, 2.10]	**<0.001**	286	1.13 [0.95, 1.34]	0.166	1.12 [0.94, 1.33]	0.201
Q5	193	1.97 [1.55, 2.53]	**<0.001**	1.90 [1.49, 2.45]	**<0.001**	322	1.29 [1.09, 1.52]	**0.003**	1.26 [1.06, 1.49]	**0.008**
*p*-trend			**<0.001**		**<0.001**			**<0.001**		**0.001**

Minimally adjusted model: logistic regression model adjusted for age, age squared, sex, ethnicity. Fully adjusted model: further adjusted for TDI, smoking status, drinking status, education levels, physical activities, BMI, PRS and total energy [if the exposure was energy or energy proportion, total energy intake will not be adjusted.

UPF, ultra-processed food; CD, Crohn’s disease; UC, ulcerative colitis.SD, standard deviation; OR, odds ratio; CI, confidence interval; Q, quintile; TDI, Townsend Deprivation Index; BMI, body mass index; PRS, polygenic risk score.

### 3.2. UPF consumption is associated with an increased risk of incidence of CD, but not UC, in individuals without a pre-existing IBD diagnosis

In the first prospective cohort study with a mean follow-up of 9.84 years (interquintile range [IQR]: 9.45-10.80), 185 849 participants without IBD were included, of whom 841 developed IBD. Of those, 251 patients were diagnosed with CD and 590 were diagnosed with UC; 101 864 participants were female [54.8%], the mean age was 56.2 [SD = 7.9], 95.9 % of participants were of White ethnicity, the mean BMI was 26.9 kg/m^2^ [SD = 4.6], and total energy intake was 8663.2 KJ [SD = 2452.4], as shown in Table 3.

**Table 3.** Characteristics and UPF intake of participants according to IBD status and subtype

	Overall [*n *= 185 849]	Non-IBD [*n* = 185008]	CD [*n* = 251]	UC [*n* = 590]
Sex [%]				
Female	101 864 [54.8]	101 457 [54.8]	120 [47.8]	287 [48.6]
Male	83 985 [45.2]	83 551 [45.2]	131 [52.2]	303 [51.4]
Age, mean [SD]	56.2 [7.9]	56.2 [7.9]	56.8 [8.1]	56.8 [7.9]
Ethnicity [%]				
White	178 299 [95.9]	177 500 [95.9]	236 [94.0]	563 [95.4]
Other	7550 [4.1]	7508 [4.1]	15 [6.0]	27 [4.6]
TDI				
High deprivation	61 950 [33.3]	61 609 [33.3]	110 [43.8]	231 [39.2]
Low deprivation	61 988 [33.4]	61 765 [33.4]	66 [26.3]	157 [26.6]
Moderate deprivation	61 911 [33.3]	61 634 [33.3]	75 [29.9]	202 [34.2]
Education level [%]				
College and above	79 013 [42.5]	78 730 [42.6]	90 [35.9]	193 [32.7]
High school and below	106 836 [57.5]	106 278 [57.4]	161 [64.1]	397 [67.3]
Smoking [%]				
Current	14 291 [7.7]	14 205 [7.7]	26 [10.4]	60 [10.2]
Never	105 753 [56.9]	105 348 [56.9]	134 [53.4]	271 [45.9]
Previous	65 805 [35.4]	65 455 [35.4]	91 [36.3]	259 [43.9]
Alcohol drinking [%]				
Current	174 051 [93.7]	173 284 [93.7]	226 [90.0]	541 [91.7]
Never	6 087 [3.3]	6 049 [3.3]	13 [5.2]	25 [4.2]
Previous	5 711 [3.1]	5 675 [3.1]	12 [4.8]	24 [4.1]
BMI, mean [SD]	26.9 [4.6]	26.9 [4.6]	27.6 [4.5]	27.5 [4.6]
Physical activity [%]				
High	62 359 [33.6]	62 089 [33.6]	87 [34.7]	183 [31.0]
Low	28 635 [15.4]	28 497 [15.4]	33 [13.1]	105 [17.8]
Middle	66 706 [35.9]	66 405 [35.9]	98 [39.0]	203 [34.4]
NA	28 149 [15.1]	28 017 [15.1]	33 [13.1]	99 [16.8]
Total energy intake KJ, mean [SD]	8663.2 [2452.4]	8662.3 [2451.8]	8729.4 [2546.3]	8914.2 [2591.8]
Number of 24-h Q				
1	66 637 [35.9]	66 295 [35.8]	111 [44.2]	231 [39.2]
2	43 973 [23.7]	43 780 [23.7]	59 [23.5]	134 [22.7]
3	40 312 [21.7]	40 158 [21.7]	42 [16.7]	112 [19.0]
4	29 345 [15.8]	29 223 [15.8]	30 [12.0]	92 [15.6]
5	5 582 [3.0]	5 552 [3.0]	9 [3.6]	21 [3.6]
Serving, mean [SD]	8.5 [4.5]	8.5 [4.5]	9.4 [4.8]	8.9 [4.6]
Serving [%]				
Q1	35 994 [19.4]	35 857 [19.4]	40 [15.9]	97 [16.4]
Q2	38 310 [20.6]	38 166 [20.6]	37 [14.7]	107 [18.1]
Q3	37 001 [19.9]	36 822 [19.9]	42 [16.7]	137 [23.2]
Q4	35 717 [19.2]	35 541 [19.2]	65 [25.9]	111 [18.8]
Q5	38 827 [20.9]	38 622 [20.9]	67 [26.7]	138 [23.4]
Energy KJ, mean [SD]	3631.9 [1816.1]	3630.9 [1815.3]	3976.9 [1913.2]	3801.1 [1989.2]
Energy KJ [%]				
Q1	37 170 [20.0]	37 012 [20.0]	43 [17.1]	115 [19.5]
Q2	37 170 [20.0]	37 029 [20.0]	41 [16.3]	100 [16.9]
Q3	37 169 [20.0]	37 012 [20.0]	40 [15.9]	117 [19.8]
Q4	37 170 [20.0]	36 983 [20.0]	61 [24.3]	126 [21.4]
Q5	37 170 [20.0]	36 972 [20.0]	66 [26.3]	132 [22.4]
Energy proportion [%], mean [SD]	41.0 [15.0]	41.0 [15.0]	45.0 [16.0]	42.0 [15.0]
Energy proportion [%]				
Q1	37 170 [20.0]	37 015 [20.0]	34 [13.5]	121 [20.5]
Q2	37 170 [20.0]	37 027 [20.0]	43 [17.1]	100 [16.9]
Q3	37 169 [20.0]	36 991 [20.0]	54 [21.5]	124 [21.0]
Q4	37 170 [20.0]	36 994 [20.0]	49 [19.5]	127 [21.5]
Q5	37 170 [20.0]	36 981 [20.0]	71 [28.3]	118 [20.0]

Energy KJ [%] in indicated participants’ number [percentage] of each quintile [Q1, Q2, Q3, Q4, Q5] of energy intake from UPFs; Q1 and Q5 were the lowest and highest quintile of the studied population, based on energy of UPF consumption, respectively. Energy proportion [%] indicated participants’ number [percentage] of each quintile [Q1, Q2, Q3, Q4, Q5] of energy percentage from UPFs.

UPF, ultra-processed food; BMI, body mass index; IBD, inflammatory bowel diseases; SD, standard deviation; TDI, Townsend Deprivation Index; CD, Crohn’s disease; UC, ulcerative colitis; NA, not available.

In the minimally adjusted model [age, age squared, sex, and ethnicity], the number of UPF servings was significantly associated with IBD risk per SD [(HR = 1.10 [95% CI: 1.03, 1.17], *p* = 0.004), for the highest quintile of UPF consumption among overall participants (HR compared with the lowest quintile = 1.34 [95% CI: 1.07, 1.67], *p* = 0.009), and for the trend [*p* = 0.001]. However, these estimates lost significance when adjusting the model further with TDI, smoking status, alcohol intake, education level, physical activity, BMI, total energy, and polygenic risk scores [Supplementary Table 3]. When analysing UPF as energy intake and energy percentage intake, respectively, the incidence per SD of UPF intake (HR = 1.09 [95% CI: 1.02, 1.16], *p* = 0.009), with trend [*p* = 0.017], and incidence per SD of intake (HR 1.07 [95% CI: 1.00, 1.15], *p* = 0.048) remained significant after considering the fully adjusted model.

When only the risk of incident CD was considered, intake of UPFs measured through servings, energy intake, or energy intake percentage were all significantly associated at the highest quintile of intake with an HR as high as 2.00 [95% CI 1.32, 3.03, *p* = 0.001] for UPF intake as a proportion of energy percentage in the fully adjusted model. In this model, the results remained significant when considering intake per SD [*p* = 0.001] and for the trend [*p* = 0.001] [Table 4].

**Table 4.** Association between UPF intake and risk of CD and UC

	CD						UC					
	Cases	Person-years	Minimally adjusted model HR 95% CI	*P*	Fully adjusted model HR 95% CI	*p*	Cases	Person-years	Minimally adjusted model HR 95% CI	*p*	Fully adjusted model HR 95% CI	*p*
Serving												
Per SD			1.17 [1.05, 1.31]	**0.005**	1.17 [1.03, 1.32]	**0.015**			1.07 [0.99, 1.16]	0.097	0.98 [0.89, 1.07]	0.629
Q1	40	355 027	ref		ref		97	355 325	ref		ref	
Q2	37	377 904	0.87 [0.56, 1.37]	0.555	0.92 [0.58, 1.44]	0.706	107	378 314	1.03 [0.78, 1.35]	0.849	0.99 [0.75, 1.31]	0.960
Q3	42	363 905	1.02 [0.66, 1.58]	0.930	1.09 [0.70, 1.70]	0.708	137	364 398	1.35 [1.04, 1.75]	**0.025**	1.26 [0.96, 1.65]	0.093
Q4	65	350 634	1.62 [1.09, 2.41]	**0.018**	1.74 [1.14, 2.65]	**0.010**	111	350 880	1.12 [0.85, 1.47]	0.424	0.99 [0.74, 1.32]	0.949
Q5	67	378 614	1.52 [1.02, 2.27]	**0.039**	1.61 [1.03, 2.51]	**0.036**	138	379 028	1.27 [0.97, 1.65]	0.081	1.01 [0.75, 1.35]	0.970
***p***-trend				**0.001**		**0.002**				0.070		0.956
Energy												
Per SD			1.18 [1.05, 1.32]	**0.006**	1.16 [1.03, 1.30]	**0.014**			1.08 [1.00, 1.17]	0.062	1.06 [0.98, 1.15]	0.127
Q1	43	365 629	ref		ref		115	365 961	ref		ref	
Q2	41	365 864	0.95 [0.62, 1.46]	0.829	0.98 [0.64, 1.50]	0.913	100	366 259	0.86 [0.66, 1.13]	0.274	0.88 [0.67, 1.15]	0.358
Q3	40	365 676	0.92 [0.60, 1.42]	0.720	0.94 [0.61, 1.46]	0.797	116	366 052	0.99 [0.76, 1.28]	0.930	1.01 [0.78, 1.31]	0.946
Q4	61	365 004	1.40 [0.94, 2.07]	0.096	1.42 [0.96, 2.10]	0.083	127	365 369	1.07 [0.83, 1.38]	0.601	1.09 [0.84, 1.41]	0.507
Q5	66	363 910	1.49 [1.01, 2.20]	**0.047**	1.46 [0.98, 2.16]	0.061	132	364 302	1.10 [0.85, 1.42]	0.475	1.08 [0.83, 1.39]	0.578
***p***-trend				**0.007**		**0.011**				0.174		0.235
Energy%												
Per SD			1.27 [1.12, 1.43]	**<0.001**	1.24 [1.09, 1.40]	**0.001**			1.03 [0.95, 1.12]	0.473	1.01 [0.93, 1.09]	0.887
Q1	34	365 164	ref		ref		121	365 452	ref		ref	
Q2	43	366 372	1.26 [0.80, 1.98]	0.312	1.28 [0.82, 2.01]	0.277	100	366 825	0.82 [0.63, 1.07]	0.136	0.83 [0.64, 1.08]	0.165
Q3	54	365 622	1.58 [1.03, 2.43]	**0.036**	1.61 [1.05, 2.48]	**0.029**	124	366 002	1.01 [0.79, 1.30]	0.936	1.02 [0.80, 1.32]	0.848
Q4	49	365 073	1.44 [0.93, 2.23]	0.106	1.45 [0.94, 2.26]	0.096	127	365 365	1.03 [0.81, 1.33]	0.789	1.03 [0.80, 1.33]	0.807
Q5	71	363 852	2.09 [1.39, 3.16]	**<0.001**	2.00 [1.32, 3.03]	**0.001**	118	364 300	0.97 [0.75, 1.25]	0.806	0.91 [0.70, 1.18]	0.473
P-trend				**<0.001**		**0.001**				0.581		0.948

Minimally adjusted model: Cox model adjusted for age, age squared, sex, ethnicity. Fully adjusted model: further adjusted for TDI, smoking status, drinking status, education levels, physical activities, BMI, PRS, and total energy [if the exposure was energy or energy proportion, total energy intake will not be adjusted]. End of follow up: 31 March 2021 for England and Scotland, and 28 February 2018 for Wales.

UPF, ultra-processed food; IBD, inflammatory bowel diseases; CD, Crohn’s disease; UC, ulcerative colitis.SD, standard deviation; HR, hazard ratio; CI, confidence interval; TDI, Townsend Deprivation Index; BMI, body mass index; PRS, polygenic risk score; ref, reference value.

For UC, however, we did not find any significant associations between UPF consumption and risk for developing UC [Table 4].

### 3.3. Association between UPF consumption in IBD patients and disease outcomes

In the second prospective cohort study, 2005 IBD patients were included, of whom 680 had a diagnosis of CD and 1325 of UC; 1026 of the included participants were female [51.2%], the mean age was 56.7 [SD = 7.9], 1939 were of White ethnicity [96.7%], the mean BMI was 26.7 kg/m^2^ [SD = 4.5], and the mean total energy intake was 8963.7 KJ [SD = 2536.3], as shown in Table 5.

**Table 5.** Characteristics and UPF intake of IBD participants

	IBD [*n* = 2005]	CD [*n* = 680]	UC [*n* = 1325]
Sex [%]			
Female	1026 [51.2]	370 [54.4]	656 [49.5]
Male	979 [48.8]	310 [45.6]	669 [50.5]
Age, mean [SD]	56.7 [7.9]	55.7 [8.0]	57.2 [7.8]
Ethnicity [%]			
White	1939 [96.7]	658 [96.8]	1281 [96.7]
Other	66 [3.3]	22 [3.2]	44 [3.3]
TDI			
High deprivation	668 [33.3]	236 [34.7]	432 [32.6]
Low deprivation	669 [33.4]	237 [34.9]	432 [32.6]
Moderate deprivation	668 [33.3]	207 [30.4]	461 [34.8]
Education level [%]			
College and above	725 [36.2]	234 [34.4]	491 [37.1]
High school and below	1280 [63.8]	446 [65.6]	834 [62.9]
Smoking [%]			
Current	125 [6.2]	62 [9.1]	63 [4.8]
Never	963 [48.0]	314 [46.2]	649 [49.0]
Previous	917 [45.7]	304 [44.7]	613 [46.3]
Alcohol drinking [%]			
Current	1835 [91.5]	613 [90.1]	1222 [92.2]
Never	77 [3.8]	28 [4.1]	49 [3.7]
Previous	93 [4.6]	39 [5.7]	54 [4.1]
BMI, mean [SD]	26.7 [4.5]	26.3 [4.4]	26.9 [4.6]
Physical activity [%]			
High	595 [29.7]	197 [29.0]	398 [30.0]
Low	373 [18.6]	143 [21.0]	230 [17.4]
Middle	698 [34.8]	224 [32.9]	474 [35.8]
NA	339 [16.9]	116 [17.1]	223 [16.8]
Total energy intake KJ, mean [SD]	8963.7 [2536.3]	9082.3 [2596.8]	8902.8 [2503.5]
Number of 24-h Q			
1	789 [39.4]	275 [40.4]	514 [38.8]
2	455 [22.7]	154 [22.6]	301 [22.7]
3	417 [20.8]	134 [19.7]	283 [21.4]
4	289 [14.4]	101 [14.9]	188 [14.2]
5	55 [2.7]	16 [2.4]	39 [2.9]
Serving, mean [SD]	9.3 [5.0]	9.8 [5.1]	9.0 [4.9]
Serving [%]			
Q1	399 [19.9]	111 [16.3]	288 [21.7]
Q2	386 [19.3]	110 [16.2]	276 [20.8]
Q3	417 [20.8]	162 [23.8]	255 [19.2]
Q4	387 [19.3]	140 [20.6]	247 [18.6]
Q5	416 [20.7]	157 [23.1]	259 [19.5]
Energy KJ, mean [SD]	3952.9 [1910.6]	4131.1 [2006.8]	3861.4 [1853.5]
Energy KJ [%]			
Q1	401 [20.0]	126 [18.5]	275 [20.8]
Q2	401 [20.0]	122 [17.9]	279 [21.1]
Q3	401 [20.0]	131 [19.3]	270 [20.4]
Q4	401 [20.0]	146 [21.5]	255 [19.2]
Q5	401 [20.0]	155 [22.8]	246 [18.6]
Energy proportion [%], mean [SD]	44.0 [16.0]	45.0 [16.0]	43.0 [16.0]
Energy proportion [%]			
Q1	401 [20.0]	111 [16.3]	290 [21.9]
Q2	401 [20.0]	133 [19.6]	268 [20.2]
Q3	401 [20.0]	148 [21.8]	253 [19.1]
Q4	401 [20.0]	139 [20.4]	262 [19.8]
Q5	401 [20.0]	149 [21.9]	252 [19.0]
Age at diagnosis, years [SD]	41.0 [14.2]	39.2 [14.7]	41.9 [13.9]

Energy KJ [%] in indicated participants’ number [percentage] of each quintile [Q1, Q2, Q3, Q4, Q5] of energy intake from UPFs; Q1 and Q5 were the lowest and highest quintile of the studied population, based on energy of UPF consumption, respectively. Energy proportion [%] in indicated participants’ number [percentage] of each quintile [Q1, Q2, Q3, Q4, Q5] of energy percentage from UPFs.

UPF, ultra-processed food; BMI, body mass index; IBD, inflammatory bowel diseases; SD, standard deviation; TDI, Townsend Deprivation Index; CD, Crohn’s disease; UC, ulcerative colitis; NA, not available.

#### 3.3.1. Need for surgery in IBD

Regarding the association between UPF intake and risk of IBD-related operations in the fully adjusted model [which was further expanded with the additional high-risk clinical features of age at diagnosis, disease location and duration, medication use, stricturing and penetrating behaviour for CD, and baseline fever and weight loss for UC],^29–31^ we found significant results for all measures of UPF intake for the highest quintile of consumption among IBD patients, per SD and for the trend [Supplementary Table 4]. Furthermore there was a clear dose-response relationship present, with the highest HR of 4.06 [95% CI 1.52, 10.86, *p* = 0.005] for UPF intake as energy intake for the fifth quintile in the fully adjusted model.

When assessing the risk for CD and UC separately, the effect seemed to be driven by UC patients with an HR of 3.25 [95% CI 1.12, 9.44, *p* = 0.030] for the highest quintile of UPF servings among UC patients [Table 6]. However, it needs to be noted that cases were few and the results on risk for colectomy in both CD and UC were less consistent [Supplementary Tables 5 and Table 7].

**Table 6.** Association between UPF intake and risk of IBD-related operations in CD, UC patients

	CD						UC					
	Cases	Person-years	Minimally adjusted model HR 95% CI	*P*	Fully adjusted model HR 95% CI	*p*	Cases	Person-years	Minimally adjusted model HR 95% CI	*p*	Fully adjusted model HR 95% CI	*p*
Serving												
Per SD			1.17 [0.88, 1.57]	0.284	1.24 [0.87, 1.77]	0.233			1.15 [0.94, 1.41]	0.164	1.06 [0.84, 1.34]	0.637
Q1	4	1038	ref		ref		6	2279	ref		ref	
Q2	3	947	0.79 [0.18, 3.52]	0.752	0.75 [0.16, 3.58]	0.721	15	2195	2.41 [0.93, 6.25]	0.069	2.76 [0.95, 8.02]	0.062
Q3	11	1058	2.69 [0.85, 8.46]	0.091	2.57 [0.76, 8.68]	0.128	25	2379	3.71 [1.51, 9.13]	**0.004**	4.75 [1.73, 13.05]	**0.003**
Q4	12	900	3.44 [1.11, 10.70]	**0.033**	3.65 [1.04, 12.73]	**0.043**	13	2321	2.09 [0.79, 5.53]	0.138	1.69 [0.54, 5.28]	0.366
Q5	7	1097	1.62 [0.47, 5.57]	0.440	1.89 [0.47, 7.57]	0.369	24	2490	3.46 [1.40, 8.56]	**0.007**	3.25 [1.12, 9.44]	**0.030**
***p***-trend				0.105		0.078				**0.031**		0.245
Energy												
Per SD			1.30 [0.97, 1.76]	0.079	1.29 [0.94, 1.76]	0.116			1.28 [1.05, 1.57]	**0.016**	1.27 [1.02, 1.58]	**0.030**
Q1	3	1033	ref		ref		7	2380	ref		ref	
Q2	11	1016	3.75 [1.05, 13.45]	**0.042**	4.25 [1.16, 15.49]	**0.028**	14	2322	2.29 [0.92, 5.71]	0.075	2.14 [0.79, 5.82]	0.135
Q3	4	1012	1.40 [0.31, 6.29]	0.658	1.28 [0.28, 5.79]	0.752	22	2301	3.07 [1.30, 7.21]	**0.010**	2.71 [1.06, 6.96]	**0.038**
Q4	8	1011	2.70 [0.71, 10.19]	0.144	2.85 [0.72, 11.28]	0.136	19	2335	2.50 [1.04, 6.00]	**0.040**	2.61 [1.03, 6.62]	**0.044**
Q5	11	967	4.09 [1.12, 14.86]	**0.033**	4.45 [1.17, 16.89]	**0.028**	21	2328	2.83 [1.19, 6.72]	**0.018**	2.54 [1.01, 6.40]	**0.048**
***p***-trend				0.098		0.103				**0.037**		0.069
Energy%												
Per SD			1.35 [0.97, 1.87]	0.071	1.33 [0.94, 1.88]	0.110			1.29 [1.04, 1.60]	**0.020**	1.31 [1.04, 1.66]	**0.024**
Q1	4	1025	ref		ref		8	2359	ref		ref	
Q2	8	999	2.08 [0.62, 6.93]	0.233	2.25 [0.67, 7.60]	0.191	13	2351	1.60 [0.66, 3.86]	0.299	1.59 [0.59, 4.34]	0.361
Q3	6	1035	1.43 [0.40, 5.08]	0.579	1.36 [0.38, 4.94]	0.638	19	2328	2.39 [1.04, 5.47]	**0.040**	2.26 [0.87, 5.91]	0.096
Q4	9	988	2.41 [0.74, 7.85]	0.144	2.94 [0.87, 9.91]	0.082	22	2323	2.54 [1.13, 5.71]	**0.025**	2.76 [1.10, 6.90]	**0.030**
Q5	10	992	2.56 [0.80, 8.19]	0.113	2.47 [0.74, 8.21]	0.142	21	2305	2.63 [1.16, 5.95]	**0.020**	2.84 [1.13, 7.13]	**0.026**
***p***-trend				0.118		0.137				**0.009**		**0.009**

Minimally adjusted model: Cox model adjusted for age, age squared, sex, ethnicity. Fully adjusted model: further adjusted for TDI, smoking status, drinking status, education levels, physical activities, BMI, PRS, total energy [if the exposure was energy or energy proportion, total energy intake will not be adjusted], age at diagnosis, disease location, disease duration, medication use, disease behaviour [stricturing and penetrating behaviour] [only for CD], baseline fever and weight loss [only for UC].

End of follow-up: 31 March 2021 for England and Scotland, 28 February 2018 for Wales.

UPF, ultra-processed food; IBD, inflammatory bowel diseases; CD, Crohn’s disease; UC, ulcerative colitis.SD, standard deviation; HR, hazard ratio; CI, confidence interval; ref, reference value; TDI, Townsend Deprivation Index; BMI, body mass index; PRS, polygenic risk score.

**Table 7.** Association between UPF intake and risk of colectomy in CD, UC patients

	CD						UC					
	Cases	Person-years	Minimally adjusted model HR 95% CI	*p*	Fully adjusted model HR 95% CI	*p*	Cases	Person-years	Minimally adjusted model HR 95% CI	*p*	Fully adjusted model HR 95% CI	*p*
Serving												
Per SD			1.21 [0.89, 1.65]	0.217	1.37 [0.93, 2.02]	0.108			1.15 [0.85, 1.57]	0.362	1.09 [0.75, 1.60]	0.646
Q1	2	1106	ref		ref		2	2303	ref		ref	
Q2	3	1004	1.57 [0.26, 9.43]	0.620	1.47 [0.23, 9.21]	0.683	6	2173	2.95 [0.59, 14.64]	0.186	2.81 [0.54, 14.58]	0.219
Q3	8	1100	4.07 [0.86, 19.17]	0.076	3.66 [0.74, 18.16]	0.112	13	2406	5.51 [1.23, 24.65]	**0.026**	5.41 [1.19, 24.66]	**0.029**
Q4	13	1083	6.71 [1.51, 29.79]	**0.012**	7.66 [1.55, 37.78]	**0.012**	4	2320	1.89 [0.34, 10.39]	0.465	1.29 [0.20, 8.22]	0.788
Q5	5	1091	2.47 [0.48, 12.79]	0.280	3.41 [0.57, 20.50]	0.180	11	2459	4.75 [1.04, 21.77]	**0.045**	3.41 [0.67, 17.32]	0.139
***p***-trend				0.053		**0.022**				0.122		0.403
Energy												
Per SD			1.33 [0.97, 1.83]	0.076	1.32 [0.95, 1.83]	0.093			1.18 [0.86, 1.62]	0.295	1.16 [0.83, 1.63]	0.381
Q1	1	1096	ref		ref		4	2376	ref		ref	
Q2	9	1107	9.03 [1.14, 71.30]	**0.037**	9.20 [1.15, 73.86]	**0.037**	7	2325	1.82 [0.53, 6.26]	0.345	1.71 [0.48, 6.04]	0.404
Q3	6	1057	6.41 [0.77, 53.38]	0.086	6.49 [0.77, 54.61]	0.085	10	2303	2.47 [0.77, 7.95]	0.129	1.84 [0.53, 6.32]	0.335
Q4	7	1081	7.13 [0.88, 58.08]	0.066	7.42 [0.89, 61.66]	0.064	7	2320	1.58 [0.46, 5.44]	0.471	1.61 [0.45, 5.70]	0.463
Q5	8	1044	8.90 [1.10, 72.00]	**0.040**	9.56 [1.14, 79.83]	**0.037**	8	2339	1.79 [0.53, 6.03]	0.345	1.52 [0.44, 5.20]	0.505
***p***-trend				0.096		0.089				0.532		0.664
Energy%												
Per SD			1.36 [0.95, 1.94]	0.093	1.44 [0.98, 2.13]	0.065			1.21 [0.88, 1.68]	0.245	1.21 [0.84, 1.74]	0.305
Q1	2	1090	ref		ref		4	2361	ref		ref	
Q2	8	1074	4.04 [0.86, 19.10]	0.078	4.11 [0.86, 19.72]	0.077	8	2338	2.09 [0.63, 6.99]	0.231	1.56 [0.45, 5.43]	0.481
Q3	6	1098	2.85 [0.57, 14.12]	0.200	2.95 [0.59, 14.81]	0.190	5	2317	1.21 [0.32, 4.52]	0.778	0.82 [0.20, 3.34]	0.777
Q4	7	1064	3.75 [0.78, 18.09]	0.100	4.58 [0.91, 22.93]	0.064	8	2351	1.89 [0.57, 6.31]	0.298	1.58 [0.47, 5.34]	0.463
Q5	8	1057	4.07 [0.86, 19.23]	0.076	4.23 [0.86, 20.69]	0.075	11	2296	2.60 [0.83, 8.19]	0.103	2.28 [0.71, 7.36]	0.168
***p***-trend				0.147		0.116				0.146		0.164

Minimally adjusted model: Cox model adjusted for age, age-squared, sex, ethnicity. Fully adjusted model: further adjusted for TDI, smoking status, drinking status, education levels, physical activities, BMI, PRS, and total energy [if the exposure was energy or energy proportion, total energy intake will not be adjusted], age at diagnosis, disease location and duration, medication use, disease behaviour [stricturing and penetrating behaviour] [only for CD], baseline fever and weight loss [only for UC].

End of follow-up: 31 March 2021 for England and Scotland, 28 February 2018 for Wales.

UPF, ultra-processed food; IBD, inflammatory bowel diseases; CD, Crohn’s disease; UC, ulcerative colitis.SD, standard deviation; HR, hazard ratio; CI, confidence interval; ref, reference value; TDI, Townsend Deprivation Index; BMI, body mass index; PRS, polygenic risk score.

#### 3.3.2. colorectal neoplasia in IBD

When assessing possible associations between UPF intake and risk of benign colorectal neoplasm in CD, we found a signal for the number of UPF servings (HR for the highest quintile of 3.21 [95% CI 1.15, 8.98, *p* = 0.026], and *p* = 0.010 for the trend in the fully adjusted model) [Table 8]. Although a dose-response relationship was clear and results showed significance, only 65 cases were observed and curiously, results were only significant for the fully adjusted and not for the minimally adjusted model.

**Table 8.** Association between UPF intake and risk of benign colorectal neoplasm in CD, UC patients

	CD						UC					
	Cases	Person-years	Minimally adjusted model HR 95% CI	*P*	Fully adjusted model HR 95% CI	*p*	Cases	Person-years	Minimally adjusted model HR 95% CI	*p*	Fully adjusted model HR 95% CI	*p*
Serving												
Per SD			1.10 [0.87, 1.39]	0.414	1.28 [0.98, 1.66]	0.068			1.02 [0.89, 1.17]	0.782	0.98 [0.82, 1.16]	0.796
Q1	8	1114	ref		ref		35	1898	ref		ref	
Q2	8	984	1.14 [0.43, 3.03]	0.800	1.40 [0.49, 3.98]	0.532	46	1819	1.33 [0.85, 2.07]	0.207	1.16 [0.72, 1.86]	0.551
Q3	17	1134	2.02 [0.87, 4.69]	0.101	2.59 [1.03, 6.52]	**0.043**	35	2230	0.73 [0.45, 1.18]	0.199	0.72 [0.43, 1.19]	0.200
Q4	17	1174	1.81 [0.78, 4.21]	0.168	2.76 [1.07, 7.12]	**0.035**	33	1963	0.82 [0.51, 1.32]	0.414	0.63 [0.37, 1.08]	0.094
Q5	15	1090	1.66 [0.70, 3.94]	0.249	3.21 [1.15, 8.98]	**0.026**	51	2039	1.13 [0.73, 1.77]	0.576	0.95 [0.57, 1.58]	0.833
***p***-trend				0.166		**0.010**				0.772		0.313
Energy												
Per SD			0.94 [0.73, 1.21]	0.651	0.98 [0.75, 1.29]	0.911			1.04 [0.90, 1.20]	0.590	1.04 [0.90, 1.21]	0.573
Q1	10	1090	ref		ref		41	2001	ref		ref	
Q2	14	1145	1.36 [0.60, 3.07]	0.456	1.14 [0.49, 2.67]	0.758	38	1977	0.90 [0.58, 1.40]	0.634	0.93 [0.58, 1.49]	0.759
Q3	15	1089	1.37 [0.61, 3.06]	0.445	1.33 [0.59, 3.02]	0.487	34	2017	0.77 [0.49, 1.22]	0.261	0.77 [0.47, 1.26]	0.305
Q4	14	1097	1.25 [0.55, 2.82]	0.593	1.21 [0.52, 2.84]	0.660	35	2078	0.68 [0.43, 1.08]	0.100	0.68 [0.42, 1.12]	0.134
Q5	12	1076	1.02 [0.43, 2.40]	0.966	1.12 [0.46, 2.72]	0.802	52	1876	1.12 [0.74, 1.70]	0.595	1.09 [0.70, 1.72]	0.701
P-trend				0.918		0.786				0.931		0.987
Energy%												
Per SD			1.01 [0.79, 1.30]	0.914	1.08 [0.82, 1.42]	0.585			1.02 [0.88, 1.17]	0.830	1.00 [0.86, 1.17]	0.979
Q1	13	1088	ref		ref		42	2014	ref		ref	
Q2	12	1109	0.82 [0.37, 1.80]	0.622	0.63 [0.28, 1.44]	0.273	38	1975	0.88 [0.57, 1.38]	0.587	0.85 [0.53, 1.36]	0.489
Q3	12	1131	0.81 [0.37, 1.79]	0.608	0.82 [0.37, 1.85]	0.639	38	1994	0.85 [0.54, 1.32]	0.458	0.82 [0.51, 1.32]	0.418
Q4	15	1097	1.08 [0.51, 2.27]	0.843	1.14 [0.51, 2.55]	0.752	38	2019	0.85 [0.55, 1.32]	0.473	0.91 [0.57, 1.44]	0.675
Q5	13	1071	0.92 [0.42, 1.99]	0.829	1.01 [0.45, 2.26]	0.980	44	1948	0.99 [0.65, 1.51]	0.961	0.95 [0.60, 1.50]	0.830
***p***-trend				0.892		0.516				0.916		0.962

Minimally adjusted model: Cox model adjusted for age, age-squared, sex, ethnicity. Fully adjusted model: further adjusted for TDI, smoking status, drinking status, education levels, physical activities, BMI, PRS and total energy [if the exposure was energy or energy proportion, total energy intake will not be adjusted], age at diagnosis, disease location and duration, medication use, family history of bowel cancer, disease behaviuor [stricturing and penetrating behaviour] [only for CD], baseline fever and weight loss [only for UC].

End of follow-up: 31 March 2021 for England and Scotland, 28 February 2018 for Wales.

UPF, ultra-processed food; CD, Crohn’s disease; UC, ulcerative colitis.SD, standard deviation; HR, hazard ratio; CI, confidence interval; ref, reference value; TDI, Townsend Deprivation Index; BMI, body mass index; PRS, polygenic risk score.

No significant associations were found for UC or IBD and risk of benign colorectal neoplasm [Supplementary Table 6 and Table 8]. Similarly, no associations were found between UPF intake and risk of colorectal cancer in IBD patients, CD patients, or UC patients [Supplementary Tables 7 and Table 9].

**Table 9.** Association between UPF intake and risk of colorectal cancer in CD, UC patients

	CD						UC					
	Cases	Person-years	Minimally adjusted model HR 95% CI	P	Fully adjusted model HR 95% CI	P	Cases	Person-years	Minimally adjusted model HR 95% CI	P	Fully adjusted model HR 95% CI	P
Serving												
Per SD			0.99 [0.52, 1.91]	0.988	1.00 [0.38, 2.61]	0.996			1.07 [0.74, 1.56]	0.704	1.08 [0.70, 1.67]	0.739
Q1	1	1253	ref		ref		3	2357	ref		ref	
Q2	2	1325	2.07 [0.19, 22.97]	0.555	1.58 [0.13, 19.55]	0.719	5	2347	1.36 [0.32, 5.73]	0.672	0.92 [0.20, 4.29]	0.915
Q3	2	1126	2.13 [0.19, 23.48]	0.538	1.75 [0.13, 23.41]	0.672	4	2760	0.86 [0.19, 3.87]	0.841	0.97 [0.21, 4.52]	0.968
Q4	3	1368	2.53 [0.26, 24.39]	0.423	1.78 [0.13, 24.37]	0.668	5	2574	1.13 [0.27, 4.77]	0.872	1.35 [0.29, 6.18]	0.701
Q5	1	1320	0.82 [0.05, 13.31]	0.891	0.67 [0.02, 21.19]	0.821	8	2534	1.68 [0.43, 6.47]	0.453	1.62 [0.35, 7.39]	0.536
*p*-trend				0.995		0.943				0.494		0.393
Energy												
Per SD			0.89 [0.45, 1.76]	0.734	0.72 [0.33, 1.54]	0.391			1.05 [0.71, 1.57]	0.808	1.12 [0.75, 1.66]	0.586
Q1	0	1264	ref		ref		4	2538	ref		ref	
Q2	3	1328	-	-	-	-	8	2522	1.67 [0.50, 5.58]	0.405	2.97 [0.76, 11.54]	0.116
Q3	3	1263	-	-	-	-	4	2471	0.86 [0.21, 3.44]	0.826	1.25 [0.27, 5.76]	0.776
Q4	2	1274	-	-	-	-	3	2524	0.55 [0.12, 2.49]	0.435	0.95 [0.18, 4.92]	0.949
Q5	1	1261	-	-	-	-	6	2516	1.10 [0.30, 3.99]	0.884	1.65 [0.40, 6.91]	0.491
*p*-trend				-		-				0.517		0.848
Energy%												
Per SD			0.79 [0.41, 1.52]	0.474	0.65 [0.29, 1.46]	0.296			0.97 [0.64, 1.46]	0.876	1.07 [0.69, 1.68]	0.755
Q1	1	1267	ref		ref		5	2530	ref		ref	
Q2	4	1262	3.67 [0.41, 33.04]	0.246	2.41 [0.24, 24.26]	0.456	7	2514	1.20 [0.38, 3.80]	0.758	1.33 [0.38, 4.64]	0.651
Q3	3	1303	2.79 [0.29, 26.89]	0.375	2.68 [0.24, 29.44]	0.421	4	2492	0.67 [0.18, 2.50]	0.547	0.82 [0.20, 3.38]	0.780
Q4	0	1298	-	-	-	-	3	2545	0.50 [0.12, 2.10]	0.342	0.57 [0.12, 2.62]	0.466
Q5	1	1261	0.90 [0.06, 14.63]	0.943	0.54 [0.03, 10.63]	0.683	6	2491	1.05 [0.32, 3.49]	0.931	1.38 [0.38, 5.06]	0.626
*p*-trend				-		-				0.629		0.930

Minimally adjusted model: Cox model adjusted for age, age-squared, sex, ethnicity. Fully adjusted model: further adjusted for TDI, smoking status, drinking status, education levels, physical activities, BMI, PRS, and total energy [if the exposure was energy or energy proportion, total energy intake will not be adjusted], age at diagnosis and disease location and duration, medication use, family history of bowel cancer. disease behaviour [stricturing and penetrating behaviour] [only for CD], baseline fever and weight loss [only for UC].

End of follow-up: 31 March 2021 for England and Scotland, 28 February 2018 for Wales.

UPF, ultra-processed food; IBD, inflammatory bowel diseases; CD, Crohn’s disease; UC, ulcerative colitis; Q, quintile; SD, standard deviation; HR, hazard ratio; CI, confidence interval; ref, reference value; TDI, Townsend Deprivation Index; BMI, body mass index; PRS, polygenic risk score.

### 3.4. Subgroup and sensitivity analyses

In a subgroup analysis on the association between UPF intake and CD risk according to disease location, we found a signal for a higher risk for ileocolonic or undefined disease, but not for ileal or colonic disease [Supplementary Table 8].

When stratifying by sex, females seemed to have a significantly higher risk for CD incidence when consuming higher amounts of UPFs, whereas this association was not significant for males. No significant effect modification by sex was observed in these associations [Supplementary Table 9–14].

In a sensitivity analysis, we obtained similar results for the risk of CD and UC incidence when excluding liquid UPF foods. We also obtained similar results when further adjusting for nutrients intake [total fat, total carbohydrate, total protein, total sugar and fibre, saturated fat, and polyunsaturated fat intake], AHEI, urine sodium, CCI, CRP, and when excluding participants with implausible energy intake and participants with incident CD or UC within the first year of recruitment. When using multiple imputation to process covariates, again, similar results were obtained, supporting the robustness of our results [Supplementary Table 15–21].

## 4. Discussion

In this large cross-sectional and prospective cohort study with 187 854 participants, we provide evidence that UPF intake is higher in individuals with a pre-existing diagnosis of IBD than in other individuals, followed in the UK biobank. Furthermore, we report a significant association between UPF consumption and an increased risk of incident CD, but not UC, in individuals without a pre-existing IBD diagnosis. We also report that increased intake of UPFs might contribute to an increased need for surgery and an increased risk of benign colorectal neoplasia in patients with an IBD diagnosis.

Most importantly, we report a robust and significant association between higher UPF intake and an increased incidence of CD [HR of 2.00], but not UC, although more interventional studies are needed to explore any causal effect. A different methodology was used in our strategy to capture dietary intakes (namely 24-h dietary recall as opposed to food frequency questionnaires [FFQs]), but consistent with those from the Nurses’ Health study from Lo *et al.* and the PURE cohort from Narula *et al.* in the association between UPF intake and CD risk [Supplementary Table 22].^12,14^ However, we were unable to replicate the signal for UC incidence reported in the unadjusted analysis by Narula *et al*. In fact, it is noteworthy that in their fully adjusted model, this signal failed to reach statistical significance. Nonetheless, possible demographic explanations for the apparent inconsistency between these datasets may be considered worthy of discussion. These include the effect of the slightly younger cohort and multiple ethnicities and regions that were explored in the PURE cohort, which we were unable to capture through UK Biobank which focuses on middle-aged adults. It could be possible that UPF intake exerts a differential effect in different age groups, or that the cumulative UPF intake in one’s lifetime should be considered as well. In addition, we cannot exclude the possibility that UPF intake interacts with [epi]genetic predispositions that certainly vary between different ethnicities, which we were unable to correct for as this study was performed on UK data only.

Given the demographic of UK Biobank, we are unable to draw conclusions regarding the possible association of IBD incidence and UPF intake in the paediatric and younger adult population. Nonetheless, the highly significant association between UPF intake and risk of incident CD strengthens our conclusion that CD, but not UC, incidence is associated with UPF intake. This apparently exclusive association between UPF and CD might relate to a greater biological propensity of CD to react to luminal contents in the gut [such as faecal derivatives and nutrients], as evidenced by the efficacy of exclusive enteral nutrition in paediatric CD and the role of diversion of the faecal stream in controlling inflammation in CD.^32,33^ Mechanistically, evidence from *in vitro*, animal, and human trials is emerging to understand how UPFs might drive gut inflammation. As an illustration, certain food additives that are frequently found in UPFs were reported to affect permeability of epithelial cell cultures,^34^ induce intestinal inflammation in susceptible mice, and give rise to colonic ulcerations in guinea pigs, resembling those in humans, when administered through their drinking water.^9,35,36^ Moreover, a study assessing the effect of another food additive [namely carboxymethylcellulose] in healthy volunteers found a perturbation in the faecal microbiota, with a reduced diversity and a decrease in short chain fatty acids.^37^

The study performed by Vasseur *et al*. in France did not show any relationship between UPF intake and IBD incidence.^13^ However, since this prospective cohort using 24-h dietary recall was only able to capture 75 cases over their follow-up period of 2.3 years, this study was probably underpowered to detect a significant effect.

In our study, 24-h dietary recall questionnaires were used as opposed to FFQs that more broadly capture dietary habits over the past month. The latter may lack the granularity of food logs and 24-h dietary recall questionnaires that is necessary to assess UPF intake, and we therefore decided not to use FFQs in our analysis. Moreover, the FFQs available in UK Biobank are rather brief, which might differ in other studies or regions. Therefore, it is reassuring that even by using different strategies to measure dietary food intakes, consistent results on CD incidence were found.

The finding that UPF intake in patients with IBD was almost twice as high as compared with non-IBD participants is novel. However, whether the nutritional habits of these patients could have led to a higher risk of IBD incidence and those habits were maintained after diagnosis, or if patients adapted their diet after diagnosis because of gastrointestinal symptoms, cannot be ascertained and would need to be addressed in a dedicated prospective trial.

Interestingly, when analysing the disease course over time, we found a novel association between UPF intake and risk of benign colorectal neoplasia in CD, and an association between UPF intake and need for IBD-related operations in UC, raising the possibility that UPF intake might impact on IBD course and contribute to adverse events in this patient population. Intriguingly, although cases for IBD-related surgery and [benign] colorectal neoplasia were few, we did find a clear dose-response relationship. This is in line with a recently published prospective cohort study in 1133 IBD patients who were followed up for 3 years, which found a significant association between the consumption of sugar-sweetened beverages [a substantial component in UPF diets] and a decreased time to hospitalisation.^38^ Furthermore, a higher intake of these beverages was also associated with disease severity biomarkers and inflammation. Notably—and relevant to our findings—a higher intake of sugar-sweetened beverages in adulthood and adolescence was associated with a higher risk of early-onset colorectal cancer among women.^39^ Of course, the same biological explanation as to how UPFs might drive intestinal inflammation discussed above, might also contribute to an unfavourable disease course and complications.

Although evidence from cohort studies and laboratory work supported the current findings of UPF intake and IBD-related adverse outcomes, the UPF components such as food additives contributing to the IBD-related outcomes remain elusive.^40,41^ A study comparing the effect of Mediterranean Diet and the Specific Carbohydrate Diet on CD did not observe any inflammation remission when consuming an elimination diet of food additives.^40^ In addition, another study that investigated the exclusive enteral nutrition formulas used for the management of CD, reported that food additives are common ingredients in these nutrition feeds.^41^ Thus, the role UPFs in managing IBD is ought to be explored in detail by interventional studies and laboratory experiments.

Our study has several strengths. To our knowledge, this is the first study to investigate the associations between IBD and UPF intake, using different measurements in a cross-sectional fashion, and to study the influence of UPFs on IBD-relevant outcomes. We were also able to correct for possibly important confounding variables such as social deprivation, BMI, comorbidities, and genetic risk. In particular, genetic factors are thought to contribute significantly to the development of IBD,^21^ and we made efforts in this study to minimise the influence of genetic susceptibility by adjusting for polygenic risk score. Furthermore, this is a very large cohort study with a similar number of incident IBD cases [841 cases] as compared with the 857 cases of the Nurses’ Health study cohort which remains the study with the largest incident cases today looking at UPFs.^14^

We acknowledge, however, certain limitations of our study. First, the 24-h dietary recall was only captured once for several participants, making it impossible to account for possible changes in dietary habits of the participants over time. Next, we cannot exclude the possibility that participants adjusted their diet due to gastrointestinal symptoms that later turned out to be caused by new-onset IBD. However a sensitivity analysis, excluding all cases with diagnoses within 1 year after recruitment, yielded similar results, suggesting that this potential phenomenon did not influence our results. Last, it is important to note that UPF refers to the method of [industrial] food processing and not a specific food item per se. Consequently, these types of food products are also typically high-energy-dense products, high in sugar, unhealthy fats, and salt, and low in dietary fibre, protein, vitamins, and minerals.^2^ In addition, colours, flavours, emulsifiers, and other additives are frequently added to make the final product palatable or hyper-palatable.^3^ In this study, we adjusted the models for total energy intake, nutrient intake [total fat, carbohydrate, protein intake], and urine sodium, but we were unable to tease out the potential role of food additives separately. The cut-off for misreported energy was set empirically without considering the basal metabolic rate [BMR] and the proportion of individuals whose energy intake below 1.1 × BMR-500 kcal [calculate BMR using the Henry equation]^42^ was 5% in the current study. Also, as the UK McCance and Widdowson’s food compositions reference from 2002 was used to calculate nutritional values, it is probable that in the past 20 years these estimates have become less accurate. Further laboratory and clinical research aimed at these compounds specifically will be critical to determine their role in driving IBD risk and outcomes.

In conclusion, in this nationwide cross-sectional and prospective cohort study of over 180 000 participants, we report an association between UPF consumption and incidence of CD, but not of UC. Furthermore, we found that UPF intake is higher in IBD patients than in non-IBD controls and that this might impact on disease outcomes. Taken together, we provide further evidence to implicate UPFs in the development and disease course of IBD, which might represent a promising strategy in tackling its globally increasing incidence. Further mechanistic and epidemiological research will be needed to further understand the biological basis for these findings and the impact of UPF intake in the developed and undeveloped worlds. Last, the influence of UPF on IBD incidence in all age groups will need further consideration, most notably in areas where IBD incidence in the paediatric population is increasing rapidly.

Researchers can request the data we used and approval from the UK Biobank at: [www.ukbiobank.ac.uk/].

## Supplementary Material

jjac167_suppl_Supplementary_Tables
