# Supplementary material for: Intake of Ultra-processed Foods Is Associated with an Increased Risk of Crohn’s Disease: A Cross-sectional and Prospective Analysis of 187 154 Participants in the UK Biobank
Source: J Crohns Colitis. 2022 Oct 28;17(4):535–52. doi: 10.1093/ecco-jcc/jjac167 (PMC10115229; doi:10.1093/ecco-jcc/jjac167)
Supplement: jjac167_suppl_Supplementary_Tables [file jjac167_suppl_supplementary_tables.docx]

Intake of ultra-processed foods is associated with an increased risk of Crohn’s disease: a cross-sectional and prospective analysis of 187,154 participants in the UK Biobank

Jie Chen, Judith Wellens, Kalla Rahul, Tian Fu, Minzi Deng, Han Zhang, Shuai Yuan, Xiaoyan Wang, Evropi Theodoratou, Xue Li, Jack Satsangi

Supplementary tables

[Supplementary table 1. UPF items in the UK Biobank 3](#_Toc115211551)

[Supplementary table 2. Association between UPF intake and IBD 6](#_Toc115211552)

[Supplementary table 3. Association between UPF intake and risk of IBD 7](#_Toc115211553)

[Supplementary table 4. Association between UPF intake and risk of IBD-related operations in IBD patients 8](#_Toc115211554)

[Supplementary table 5. Association between UPF intake and risk of colectomy in IBD patients 9](#_Toc115211555)

[Supplementary table 6. Association between UPF intake and risk of benign colorectal neoplasm in IBD patients 10](#_Toc115211556)

[Supplementary table 7. Association between UPF intake and risk of colorectal cancer in IBD patients 11](#_Toc115211557)

[Supplementary table 8. Association between UPF intake and CD risk according to diseases location 12](#_Toc115211558)

[Supplementary table 9. Subgroup analyses for the association between UPF intake and risk of CD and UC stratified by age 13](#_Toc115211559)

[Supplementary table 10. Subgroup analyses for the association between UPF intake and risk of CD and UC stratified by sex 14](#_Toc115211560)

[Supplementary table 11. Subgroup analyses for the association between UPF intake and risk of CD and UC stratified by alcohol drinking status 15](#_Toc115211561)

[Supplementary table 12. Subgroup analyses for the association between UPF intake and risk of CD and UC stratified by smoking status 16](#_Toc115211562)

[Supplementary table 13. Subgroup analyses for the association between UPF intake and risk of CD and UC stratified by BMI 17](#_Toc115211563)

[Supplementary table 14. Subgroup analyses for the association between UPF intake and risk of CD and UC stratified by PRS 18](#_Toc115211564)

[Supplementary table 15. Sensitivity analyses for the association between UPF intake and risk of CD and UC when excluding liquid UPF food 19](#_Toc115211565)

[Supplementary table 16. Sensitivity analyses for the association between UPF intake and risk of CD and UC when further adjusting for nutrients intake and AHEI 20](#_Toc115211566)

[Supplementary table 17. Sensitivity analyses for the association between UPF intake and risk of CD and UC when further adjusting for urine sodium 22](#_Toc115211567)

[Supplementary table 18. Sensitivity analyses for the association between UPF intake and risk of CD and UC when further adjusting for Charlson Comorbidity Index 23](#_Toc115211568)

[Supplementary table 19. Sensitivity analyses for the association between UPF intake and risk of CD and UC when further adjusting for C-reactive protein 24](#_Toc115211569)

[Supplementary table 20. Sensitivity analyses for the association between UPF intake and risk of CD and UC when excluding participants with implausible energy intake or excluding participants with or incident CD or UC within the first year 25](#_Toc115211570)

[Supplementary table 21. Sensitivity analyses for the association between UPF intake and risk of CD and UC when using multiple imputation to process covariate 27](#_Toc115211571)

[Supplementary table 22. Characteristics of summarized studies of associations between UPF intake and risk of IBD 28](#_Toc115211572)

# Supplementary table 1. UPF items in the UK Biobank

| **Data field** | **Food item** | **Portion size**  **g/serving ^a^** | **Energy**  **KJ/100 g ^b^** |
| --- | --- | --- | --- |
| 100160 | Low calorie drink intake | 330 | 43 |
| 100170 | Fizzy drink intake carbonated (fizzy) drinks | 330 | 174 |
| 100180 | Squash intake | 250 | 159 |
| 100250 | Instant coffee intake | 190 | 8 |
| 100380 | Intake of artificial sweetener added to coffee ^c^ | 6 | 200 |
| 100500 | Intake of artificial sweetener added to tea ^d^ | 6 | 200 |
| 100530 | Flavoured milk intake | 250 | 270 |
| 100720 | Fortified wine intake | 50 | 481 |
| 100730 | Spirits intake | 23 | 919 |
| 100770 | Porridge intake | 203.5 | 195 |
| 100800 | muesli intake | 100 | 1540 |
| 100810 | Oat crunch intake | 100 | 1639 |
| 100820 | Sweetened cereal intake | 38 | 1632 |
| 100830 | Plain cereal intake | 30 | 1601 |
| 100840 | Bran cereal intake | 50 | 1406 |
| 100850 | Whole-wheat cereal intake | 44 | 1474 |
| 100860 | Other cereal intake | 44 | 1566 |
| 101090 | Bap intake | 90 | 1065 |
| 101160 | Bread roll intake | 60 | 1084 |
| 101230 | Naan bread intake | 160 | 1206 |
| 101250 | Crispbread intake | 10 | 1591 |
| 101260 | Oatcakes intake | 13 | 1737 |
| 101270 | Other bread intake | 45 | 1661 |
| 101310 | Number of bread slices with butter/margarine | Thin 10 medium 12 thick 15 | 3061 |
| 101350 | Number of baguettes with butter/margarine |  |  |
| 101390 | Number of baps with butter/margarine |  |  |
| 101430 | Number of bread rolls with butter/margarine |  |  |
| 101470 | Number of crackers/crispbreads with butter/margarine |  |  |
| 101510 | Number of oatcakes with butter/margarine |  |  |
| 101550 | Number of other bread types with butter/margarine |  |  |
| 101970 | Double crust pastry intake | 60 | 1060 |
| 101980 | Single crust pastry intake | 30 | 1310 |
| 101990 | Crumble intake | 70 | 924 |
| 102000 | Pizza intake | 150 | 1036 |
| 102010 | Pancake intake | 110 | 1065 |
| 102020 | Scotch pancake intake | 41 | 1138 |
| 102030 | Yorkshire pudding intake | 25 | 881 |
| 102040 | Indian snacks intake | 40 | 975 |
| 102050 | Croissant intake | 60 | 1563 |
| 102060 | Danish pastry intake | 110 | 1441 |
| 102070 | Scone intake | 48 | 1378 |
| 102120 | Ice-cream intake | 120 | 1229 |
| 102140 | Milk-based pudding intake | 200 | 494 |
| 102150 | Other milk-based pudding intake | 60 | 518 |
| 102170 | Soya dessert intake | 125 | 309 |
| 102180 | Fruitcake intake | 70 | 1478 |
| 102190 | Cake intake | 60 | 1617 |
| 102200 | Doughnut intake | 60 | 1414 |
| 102210 | Sponge pudding intake | 120 | 1116 |
| 102220 | Cheesecake intake | 110 | 1231 |
| 102230 | Other dessert intake | 60 | 696 |
| 102260 | Chocolate bar intake | 50 | 2081 |
| 102270 | White chocolate intake | 50 | 2212 |
| 102280 | Milk chocolate intake | 50 | 2177 |
| 102290 | Dark chocolate intake | 50 | 2273 |
| 102300 | Chocolate-covered raisin intake | 25 | 1159 |
| 102310 | Chocolate sweet intake | 36 | 1922 |
| 102320 | Diet sweets intake | 18 | 975 |
| 102330 | Sweets intake | 36 | 1793 |
| 102340 | Chocolate-covered biscuits intake | 17 | 2076 |
| 102350 | Chocolate biscuits intake | 24 | 2071 |
| 102360 | Sweet biscuits intake | 17 | 1842 |
| 102370 | Cereal bar intake | 28 | 1525 |
| 102380 | Other sweets intake sweet snacks | 40 | 1793 |
| 102460 | Crisp intake | 40 | 2186 |
| 102470 | Savoury biscuits intake | 40 | 2168 |
| 102480 | Cheesy biscuits intake | 40 | 1366 |
| 102500 | Other savoury snack intake | 40 | 2099 |
| 102530 | Powdered/instant soup intake | 200 | 270 |
| 102760 | Snackpot intake (snack pot, noodles/rice | 280 | 1541 |
| 102770 | Couscous intake | 150 | 1440 |
| 102850 | Low fat cheese spread intake | 40 | 733 |
| 102860 | Cheese spread intake | 15 | 1106 |
| 103010 | Sausage intake | 30 | 1218 |
| 103050 | Crumbed or deep-fried poultry intake (chicken or turkey in breadcrumbs or deep fried | 100 | 1111 |
| 103070 | Bacon intake | 46 | 891 |
| 103080 | Ham intake | 23 | 891 |
| 103260 | Vegetarian sausages/burgers intake | 90 | 748 |
| 103280 | Quorn intake | 90 | 389 |
| 103290 | Other vegetarian alternative intake | 90 | 1386 |
| 104000 | Baked bean intake | 135 | 335 |
| 104020 | Fried potatoes intake | 180 | 796 |
| 104050 | Mashed potato intake | 60 | 438 |

^a^ Portion size used here was according to UK McCance and Widdowson’s *The Composition of Foods 6th edition (2002) and its supplements* ^1,2^

^b^ Energy for each food used here was according to *UK McCance and Widdowson’s The Composition of Foods 6th edition (2002) and its supplements*^3^

^c^ Coffee here included Instant coffee (100250), Filtered coffee (100270), Cappuccino (100290), Latte (100300), Espresso (100310), Other coffee type (100330)

^d^ Tea here included Standard tea (100400), Rooibos tea (100410), Green tea (100420), Herbal tea (100430), Other tea (100440)

## Supplementary table 2. Association between UPF intake and IBD

|  | **IBD Cases** | **Minimally adjusted model**  **OR 95%CI** | **P** | **Fully**  **adjusted model**  **OR 95%CI** | **P** |
| --- | --- | --- | --- | --- | --- |
| Serving |  |  |  |  |  |
| Per SD |  | 1.16 (1.11, 1.21) | **<0.001** | 1.12 (1.07, 1.17) | **<0.001** |
| Q1 | 339 | ref |  | ref |  |
| Q2 | 351 | 0.96 (0.83, 1.12) | 0.611 | 0.94 (0.80, 1.09) | 0.393 |
| Q3 | 341 | 0.95 (0.82, 1.11) | 0.515 | 0.91 (0.78, 1.06) | 0.227 |
| Q4 | 434 | 1.27 (1.10, 1.47) | **0.001** | 1.18 (1.02, 1.38) | **0.031** |
| Q5 | 540 | 1.43 (1.24, 1.64) | **<0.001** | 1.28 (1.10, 1.50) | **0.002** |
| P-trend |  |  | **<0.001** |  | **<0.001** |
| Energy |  |  |  |  |  |
| Per SD |  | 1.17 (1.12, 1.22) | **<0.001** | 1.17 (1.12, 1.22) | **<0.001** |
| Q1 | 331 | ref |  | ref |  |
| Q2 | 324 | 0.97 (0.83, 1.13) | 0.707 | 0.98 (0.84, 1.15) | 0.812 |
| Q3 | 376 | 1.12 (0.97, 1.30) | 0.124 | 1.13 (0.97, 1.31) | 0.108 |
| Q4 | 451 | 1.35 (1.17, 1.56) | **<0.001** | 1.35 (1.17, 1.56) | **<0.001** |
| Q5 | 523 | 1.56 (1.36, 1.80) | **<0.001** | 1.56 (1.35, 1.79) | **<0.001** |
| P-trend |  |  | **<0.001** |  | **<0.001** |
| Energy% |  |  |  |  |  |
| Per SD |  | 1.17 (1.12, 1.22) | **<0.001** | 1.15 (1.10, 1.20) | **<0.001** |
| Q1 | 347 | ref |  | ref |  |
| Q2 | 350 | 1.00 (0.86, 1.16) | 0.999 | 1.00 (0.86, 1.16) | 0.986 |
| Q3 | 350 | 1.00 (0.86, 1.16) | 0.965 | 1.00 (0.86, 1.16) | 0.985 |
| Q4 | 443 | 1.26 (1.10, 1.46) | **0.001** | 1.25 (1.09, 1.45) | **0.002** |
| Q5 | 515 | 1.48 (1.29, 1.70) | **<0.001** | 1.44 (1.25, 1.66) | **<0.001** |
| P-trend |  |  | **<0.001** |  | **<0.001** |

Minimally adjusted model: Logistic regression model adjusted for age, age-squared, sex, ethnicity

Fully adjusted model: further adjusted for TDI, smoking status, drinking status, education levels, physical activities, BMI, PRS and total energy (if the exposure was energy or energy proportion, total energy intake will not be adjusted. UPF, ultra-processed food; BMI, body mass index; IBD, inflammatory bowel diseases; SD, standard deviation; OR, odds ratio; CI, confidence interval.

## Supplementary table 3. Association between UPF intake and risk of IBD

|  | **Cases** | **Person-years** | **Minimally adjusted model**  **HR 95%CI** | **P** | **Fully**  **adjusted model**  **HR 95%CI** | **P** |
| --- | --- | --- | --- | --- | --- | --- |
| Serving |  |  |  |  |  |  |
| Per SD |  |  | 1.10 (1.03, 1.17) | **0.004** | 1.03 (0.96, 1.11) | 0.361 |
| Q1 | 137 | 355 564 | ref |  | ref |  |
| Q2 | 144 | 378 534 | 0.98 (0.78, 1.24) | 0.885 | 0.97 (0.77, 1.23) | 0.822 |
| Q3 | 179 | 364 654 | 1.25 (1.00, 1.57) | **0.048** | 1.22 (0.97, 1.53) | 0.094 |
| Q4 | 176 | 351 253 | 1.26 (1.01, 1.58) | **0.042** | 1.18 (0.94, 1.50) | 0.160 |
| Q5 | 205 | 379 343 | 1.34 (1.07, 1.67) | **0.009** | 1.16 (0.91, 1.48) | 0.235 |
| P-trend |  |  |  | **0.001** |  | 0.091 |
| Energy |  |  |  |  |  |  |
| Per SD |  |  | 1.11 (1.04, 1.18) | **0.002** | 1.09 (1.02, 1.16) | **0.009** |
| Q1 | 158 | 366 286 | ref |  | ref |  |
| Q2 | 141 | 366 587 | 0.89 (0.71, 1.11) | 0.297 | 0.91 (0.72, 1.14) | 0.404 |
| Q3 | 157 | 366 358 | 0.98 (0.78, 1.22) | 0.841 | 1.00 (0.80, 1.25) | 0.991 |
| Q4 | 187 | 365 643 | 1.15 (0.93, 1.43) | 0.193 | 1.17 (0.95, 1.45) | 0.144 |
| Q5 | 198 | 364 475 | 1.20 (0.97, 1.49) | 0.089 | 1.18 (0.95, 1.46) | 0.130 |
| P-trend |  |  |  | **0.010** |  | **0.017** |
| Energy% |  |  |  |  |  |  |
| Per SD |  |  | 1.10 (1.02, 1.17) | **0.008** | 1.07 (1.00, 1.15) | **0.048** |
| Q1 | 155 | 365 816 | ref |  | ref |  |
| Q2 | 143 | 367 136 | 0.91 (0.73, 1.15) | 0.444 | 0.93 (0.74, 1.17) | 0.523 |
| Q3 | 178 | 366 262 | 1.14 (0.92, 1.41) | 0.247 | 1.15 (0.93, 1.43) | 0.192 |
| Q4 | 176 | 365 660 | 1.12 (0.90, 1.39) | 0.293 | 1.12 (0.91, 1.40) | 0.289 |
| Q5 | 189 | 364 473 | 1.21 (0.98, 1.50) | 0.074 | 1.15 (0.93, 1.42) | 0.210 |
| P-trend |  |  |  | **0.016** |  | 0.064 |

Minimally adjusted model: Cox model adjusted for age, age-squared, sex, ethnicity

Fully adjusted model: further adjusted for TDI, smoking status, drinking status, education levels, physical activities, BMI, PRS and total energy (if the exposure was energy or energy proportion, total energy intake will not be adjusted. UPF, ultra-processed food; BMI, body mass index; IBD, inflammatory bowel diseases; SD, standard deviation; HR, hazard ratio; CI, confidence interval.

## Supplementary table 4. Association between UPF intake and risk of IBD-related operations in IBD patients

|  | **Cases** | **Person-years** | **Minimally adjusted model**  **HR 95%CI** | **P** | **Fully**  **adjusted model**  **HR 95%CI** | **P** |
| --- | --- | --- | --- | --- | --- | --- |
| Serving |  |  |  |  |  |  |
| Per SD |  |  | 1.17 (0.96, 1.42) | 0.111 | 1.16 (0.92, 1.46) | 0.220 |
| Q1 | 5 | 3007 | ref |  | ref |  |
| Q2 | 13 | 3508 | 2.13 (0.76, 5.98) | 0.151 | 1.98 (0.69, 5.68) | 0.204 |
| Q3 | 23 | 3154 | 4.19 (1.59, 11.04) | **0.004** | 3.93 (1.45, 10.63) | **0.007** |
| Q4 | 24 | 3233 | 4.26 (1.62, 11.21) | **0.003** | 3.88 (1.40, 10.77) | **0.009** |
| Q5 | 18 | 3345 | 3.05 (1.13, 8.27) | **0.028** | 3.06 (1.05, 8.92) | **0.040** |
| P-trend |  |  |  | **0.011** |  | **0.027** |
| Energy |  |  |  |  |  |  |
| Per SD |  |  | 1.34 (1.10, 1.62) | **0.003** | 1.32 (1.08, 1.61) | **0.007** |
| Q1 | 6 | 3319 | ref |  | ref |  |
| Q2 | 18 | 3255 | 3.00 (1.19, 7.57) | **0.020** | 3.31 (1.22, 8.96) | **0.018** |
| Q3 | 18 | 3225 | 3.01 (1.19, 7.59) | **0.020** | 2.77 (1.00, 7.65) | 0.050 |
| Q4 | 18 | 3244 | 2.99 (1.18, 7.57) | **0.021** | 3.20 (1.17, 8.70) | **0.023** |
| Q5 | 23 | 3203 | 3.87 (1.57, 9.59) | **0.003** | 4.06 (1.52, 10.86) | **0.005** |
| P-trend |  |  |  | **0.010** |  | **0.016** |
| Energy% |  |  |  |  |  |  |
| Per SD |  |  | 1.42 (1.15, 1.76) | **0.001** | 1.44 (1.14, 1.80) | **0.002** |
| Q1 | 8 | 3300 | ref |  | ref |  |
| Q2 | 17 | 3256 | 2.11 (0.91, 4.90) | 0.081 | 1.91 (0.77, 4.72) | 0.161 |
| Q3 | 13 | 3257 | 1.61 (0.67, 3.88) | 0.292 | 1.41 (0.55, 3.60) | 0.477 |
| Q4 | 20 | 3263 | 2.48 (1.09, 5.64) | **0.030** | 2.59 (1.08, 6.17) | **0.032** |
| Q5 | 25 | 3171 | 3.18 (1.43, 7.06) | **0.005** | 3.08 (1.31, 7.26) | **0.010** |
| P-trend |  |  |  | **0.004** |  | **0.004** |

Minimally adjusted model: Cox model adjusted for age, age-squared, sex, ethnicity

Fully adjusted model: further adjusted for TDI, smoking status, drinking status, education levels, physical activities, BMI, PRS and total energy (if the exposure was energy or energy proportion, total energy intake will not be adjusted), age at diagnosis, disease location and disease duration, medication use.

UPF, ultra-processed food; IBD, inflammatory bowel diseases; SD, standard deviation; HR, hazard ratio; CI, confidence interval.

## Supplementary table 5. Association between UPF intake and risk of colectomy in IBD patients

|  | **Cases** | **Person-years** | **Minimally adjusted model**  **HR 95%CI** | **P** | **Fully**  **adjusted model**  **HR 95%CI** | **P** |
| --- | --- | --- | --- | --- | --- | --- |
| Serving |  |  |  |  |  |  |
| Per SD |  |  | 1.19 (0.97, 1.48) | 0.102 | 1.19 (0.92, 1.53) | 0.191 |
| Q1 | 4 | 3113 | ref |  | ref |  |
| Q2 | 13 | 3760 | 2.58 (0.84, 7.92) | 0.098 | 2.59 (0.83, 8.06) | 0.100 |
| Q3 | 13 | 3219 | 2.96 (0.96, 9.10) | 0.059 | 2.65 (0.84, 8.36) | 0.097 |
| Q4 | 23 | 3524 | 4.84 (1.67, 14.06) | **0.004** | 4.51 (1.47, 13.81) | **0.008** |
| Q5 | 14 | 3429 | 2.98 (0.97, 9.13) | 0.056 | 2.92 (0.88, 9.68) | 0.079 |
| P-trend |  |  |  | **0.022** |  | 0.056 |
| Energy |  |  |  |  |  |  |
| Per SD |  |  | 1.27 (1.02, 1.58) | **0.034** | 1.24 (0.99, 1.56) | 0.067 |
| Q1 | 5 | 3479 | ref |  | ref |  |
| Q2 | 15 | 3415 | 3.01 (1.09, 8.30) | **0.033** | 2.63 (0.95, 7.30) | 0.063 |
| Q3 | 17 | 3346 | 3.41 (1.26, 9.27) | **0.016** | 2.74 (0.99, 7.61) | 0.053 |
| Q4 | 14 | 3416 | 2.72 (0.97, 7.60) | 0.056 | 2.37 (0.84, 6.67) | 0.103 |
| Q5 | 16 | 3390 | 3.09 (1.12, 8.52) | **0.029** | 2.63 (0.94, 7.33) | 0.064 |
| P-trend |  |  |  | 0.098 |  | 0.187 |
| Energy% |  |  |  |  |  |  |
| Per SD |  |  | 1.30 (1.02, 1.64) | **0.034** | 1.27 (0.99, 1.64) | 0.062 |
| Q1 | 6 | 3458 | ref |  | ref |  |
| Q2 | 16 | 3400 | 2.67 (1.04, 6.83) | **0.041** | 2.20 (0.85, 5.72) | 0.106 |
| Q3 | 11 | 3413 | 1.79 (0.66, 4.84) | 0.253 | 1.36 (0.49, 3.78) | 0.552 |
| Q4 | 15 | 3431 | 2.42 (0.94, 6.25) | 0.068 | 2.15 (0.83, 5.61) | 0.117 |
| Q5 | 19 | 3345 | 3.13 (1.25, 7.85) | **0.015** | 2.73 (1.07, 6.98) | **0.036** |
| P-trend |  |  |  | **0.038** |  | 0.058 |

Minimally adjusted model: Cox model adjusted for age, age-squared, sex, ethnicity

Fully adjusted model: further adjusted for TDI, smoking status, drinking status, education levels, physical activities, BMI, PRS and total energy (if the exposure was energy or energy proportion, total energy intake will not be adjusted), age at diagnosis, disease location and duration, medication use.

UPF, ultra-processed food; IBD, inflammatory bowel diseases; SD, standard deviation; HR, hazard ratio; CI, confidence interval.

## Supplementary table 6. Association between UPF intake and risk of benign colorectal neoplasm in IBD patients

|  | **Cases** | **Person-years** | **Minimally adjusted model**  **HR 95%CI** | **P** | **Fully**  **adjusted model**  **HR 95%CI** | **P** |
| --- | --- | --- | --- | --- | --- | --- |
| Serving |  |  |  |  |  |  |
| Per SD |  |  | 1.02 (0.91, 1.15) | 0.728 | 1.04 (0.90, 1.19) | 0.629 |
| Q1 | 49 | 3100 | ref |  | ref |  |
| Q2 | 54 | 3047 | 1.09 (0.74, 1.60) | 0.671 | 1.02 (0.67, 1.53) | 0.937 |
| Q3 | 50 | 3189 | 0.90 (0.60, 1.33) | 0.590 | 0.85 (0.55, 1.30) | 0.450 |
| Q4 | 46 | 3094 | 0.83 (0.55, 1.24) | 0.357 | 0.79 (0.50, 1.24) | 0.311 |
| Q5 | 66 | 3015 | 1.17 (0.80, 1.71) | 0.406 | 1.15 (0.74, 1.78) | 0.528 |
| P-trend |  |  |  | 0.809 |  | 0.817 |
| Energy |  |  |  |  |  |  |
| Per SD |  |  | 0.99 (0.88, 1.13) | 0.935 | 1.02 (0.89, 1.16) | 0.784 |
| Q1 | 51 | 3106 | ref |  | ref |  |
| Q2 | 55 | 3083 | 1.04 (0.71, 1.52) | 0.847 | 1.02 (0.68, 1.53) | 0.911 |
| Q3 | 48 | 3103 | 0.85 (0.57, 1.27) | 0.431 | 0.89 (0.58, 1.35) | 0.573 |
| Q4 | 49 | 3185 | 0.80 (0.54, 1.19) | 0.263 | 0.78 (0.52, 1.20) | 0.260 |
| Q5 | 62 | 2968 | 1.04 (0.71, 1.52) | 0.829 | 1.10 (0.74, 1.64) | 0.648 |
| P-trend |  |  |  | 0.739 |  | 0.955 |
| Energy% |  |  |  |  |  |  |
| Per SD |  |  | 1.00 (0.89, 1.14) | 0.956 | 1.00 (0.88, 1.14) | 0.973 |
| Q1 | 59 | 3082 | ref |  | ref |  |
| Q2 | 43 | 3131 | 0.66 (0.45, 0.99) | **0.042** | 0.61 (0.40, 0.93) | **0.020** |
| Q3 | 52 | 3112 | 0.82 (0.57, 1.19) | 0.302 | 0.84 (0.57, 1.23) | 0.365 |
| Q4 | 57 | 3081 | 0.89 (0.61, 1.28) | 0.516 | 0.90 (0.61, 1.32) | 0.584 |
| Q5 | 54 | 3039 | 0.86 (0.59, 1.24) | 0.413 | 0.86 (0.58, 1.27) | 0.441 |
| P-trend |  |  |  | 0.917 |  | 0.918 |

Minimally adjusted model: Cox model adjusted for age, age-squared, sex, ethnicity

Fully adjusted model: further adjusted for TDI, smoking status, drinking status, education levels, physical activities, BMI, PRS and total energy (if the exposure was energy or energy proportion, total energy intake will not be adjusted), age at diagnosis, disease location and duration, medication use, and family history of bowel cancer.

UPF, ultra-processed food; IBD, inflammatory bowel diseases; SD, standard deviation; HR, hazard ratio; CI, confidence interval.

## Supplementary table 7. Association between UPF intake and risk of colorectal cancer in IBD patients

|  | **Cases** | **Person-years** | **Minimally adjusted model**  **HR 95%CI** | **P** | **Fully**  **adjusted model**  **HR 95%CI** | **P** |
| --- | --- | --- | --- | --- | --- | --- |
| Serving |  |  |  |  |  |  |
| Per SD |  |  | 1.04 (0.75, 1.44) | 0.797 | 1.06 (0.72, 1.56) | 0.776 |
| Q1 | 5 | 3812 | ref |  | ref |  |
| Q2 | 6 | 3651 | 1.14 (0.35, 3.73) | 0.832 | 1.08 (0.31, 3.84) | 0.901 |
| Q3 | 6 | 3933 | 1.01 (0.31, 3.32) | 0.985 | 1.13 (0.33, 3.85) | 0.847 |
| Q4 | 9 | 3628 | 1.54 (0.51, 4.62) | 0.444 | 1.94 (0.60, 6.33) | 0.269 |
| Q5 | 8 | 3937 | 1.15 (0.37, 3.58) | 0.804 | 1.20 (0.33, 4.35) | 0.778 |
| P-trend |  |  |  | 0.651 |  | 0.522 |
| Energy |  |  |  |  |  |  |
| Per SD |  |  | 0.99 (0.70, 1.39) | 0.942 | 1.00 (0.70, 1.42) | 0.980 |
| Q1 | 5 | 3813 | ref |  | ref |  |
| Q2 | 10 | 3826 | 1.78 (0.61, 5.22) | 0.294 | 2.05 (0.63, 6.64) | 0.230 |
| Q3 | 6 | 3737 | 1.04 (0.32, 3.43) | 0.946 | 1.16 (0.32, 4.18) | 0.819 |
| Q4 | 6 | 3794 | 0.97 (0.29, 3.23) | 0.964 | 1.24 (0.34, 4.49) | 0.748 |
| Q5 | 7 | 3791 | 1.06 (0.33, 3.41) | 0.921 | 1.23 (0.35, 4.37) | 0.747 |
| P-trend |  |  |  | 0.605 |  | 0.769 |
| Energy% |  |  |  |  |  |  |
| Per SD |  |  | 0.91 (0.64, 1.28) | 0.579 | 0.91 (0.63, 1.33) | 0.635 |
| Q1 | 6 | 3813 | ref |  | ref |  |
| Q2 | 10 | 3777 | 1.50 (0.54, 4.14) | 0.434 | 1.69 (0.57, 5.03) | 0.348 |
| Q3 | 8 | 3785 | 1.16 (0.40, 3.36) | 0.785 | 1.23 (0.39, 3.87) | 0.721 |
| Q4 | 4 | 3833 | 0.58 (0.16, 2.05) | 0.396 | 0.64 (0.17, 2.44) | 0.515 |
| Q5 | 6 | 3753 | 0.92 (0.30, 2.87) | 0.886 | 1.04 (0.31, 3.50) | 0.949 |
| P-trend |  |  |  | 0.368 |  | 0.441 |

Minimally adjusted model: Cox model adjusted for age, age-squared, sex, ethnicity

Fully adjusted model: further adjusted for TDI, smoking status, drinking status, education levels, physical activities, BMI, PRS and total energy (if the exposure was energy or energy proportion, total energy intake will not be adjusted), age at diagnosis, disease location and duration, medication use, and family history of bowel cancer.

UPF, ultra-processed food; IBD, inflammatory bowel diseases; SD, standard deviation; HR, hazard ratio; CI, confidence interval.

# Supplementary table 8. Association between UPF intake and CD risk according to diseases location

|  | **Ileal CD** | | | |  | **Colonic CD** | | | |  | **leocolonic or location not defined CD** | | | |
| --- | --- | --- | --- | --- | --- | --- | --- | --- | --- | --- | --- | --- | --- | --- |
|  | **Cases** | **Person-years** | **HR 95%CI** | **P** |  | **Cases** | **Person-years** | **HR 95%CI** | **P** |  | **Cases** | **Person-years** | **IHR 95%CI** | **P** |
| Serving |  |  |  |  |  |  |  |  |  |  |  |  |  |  |
| Per SD |  |  | 1.19 (0.92, 1.54) | 0.190 |  |  |  | 1.02 (0.71, 1.47) | 0.904 |  |  |  | 1.19 (1.02, 1.39) | **0.025** |
| Q1 | 8 | 354 822 | Ref |  |  | 6 | 354 831 | Ref |  |  | 26 | 354 950 | Ref |  |
| Q2 | 10 | 377 241 | 1.25 (0.49, 3.19) | 0.647 |  | 6 | 377 222 | 0.92 (0.29, 2.90) | 0.890 |  | 21 | 377 310 | 0.81 (0.45, 1.45) | 0.486 |
| Q3 | 5 | 364 176 | 0.65 (0.21, 2.03) | 0.456 |  | 10 | 364 202 | 1.54 (0.54, 4.40) | 0.421 |  | 27 | 364 322 | 1.11 (0.64, 1.93) | 0.720 |
| Q4 | 12 | 350 325 | 1.60 (0.62, 4.11) | 0.332 |  | 4 | 350 283 | 0.62 (0.16, 2.32) | 0.474 |  | 49 | 350 548 | 2.11 (1.27, 3.51) | **0.004** |
| Q5 | 19 | 378 377 | 2.21 (0.87, 5.64) | 0.096 |  | 10 | 378 343 | 1.33 (0.42, 4.25) | 0.627 |  | 38 | 378 491 | 1.49 (0.85, 2.62) | 0.166 |
| P-trend |  |  |  | 0.060 |  |  |  |  | 0.873 |  |  |  |  | **0.006** |
| Energy |  |  |  |  |  |  |  |  |  |  |  |  |  |  |
| Per SD |  |  | 1.11 (0.86, 1.42) | 0.427 |  |  |  | 1.03 (0.75, 1.42) | 0.856 |  |  |  | 1.21 (1.05, 1.40) | **0.007** |
| Q1 | 9 | 365 376 | Ref |  |  | 6 | 365 360 | Ref |  |  | 28 | 365 510 | Ref |  |
| Q2 | 8 | 365 601 | 0.91 (0.35, 2.36) | 0.845 |  | 8 | 365 574 | 1.39 (0.48, 4.00) | 0.546 |  | 25 | 365 755 | 0.92 (0.54, 1.58) | 0.757 |
| Q3 | 13 | 365 391 | 1.44 (0.62, 3.39) | 0.398 |  | 5 | 365 411 | 0.87 (0.27, 2.87) | 0.822 |  | 22 | 365 589 | 0.81 (0.46, 1.42) | 0.456 |
| Q4 | 10 | 364 819 | 1.08 (0.44, 2.68) | 0.864 |  | 10 | 364 784 | 1.75 (0.63, 4.84) | 0.281 |  | 41 | 364 934 | 1.50 (0.92, 2.43) | 0.104 |
| Q5 | 14 | 363 754 | 1.37 (0.58, 3.21) | 0.475 |  | 7 | 363 751 | 1.20 (0.40, 3.60) | 0.740 |  | 45 | 363 833 | 1.59 (0.98, 2.58) | 0.059 |
| P-trend |  |  |  | 0.427 |  |  |  |  | 0.852 |  |  |  |  | **0.009** |
| Energy% |  |  |  |  |  |  |  |  |  |  |  |  |  |  |
| Per SD |  |  | 1.17 (0.90, 1.52) | 0.234 |  |  |  | 0.97 (0.70, 1.35) | 0.855 |  |  |  | 1.34 (1.15, 1.55) | **<0.001** |
| Q1 | 7 | 364 884 | Ref |  |  | 6 | 364 840 | Ref |  |  | 21 | 365 032 | Ref |  |
| Q2 | 12 | 366 097 | 1.74 (0.69, 4.43) | 0.244 |  | 7 | 366 117 | 1.19 (0.40, 3.56) | 0.749 |  | 24 | 366 299 | 1.16 (0.65, 2.09) | 0.611 |
| Q3 | 14 | 365 398 | 2.03 (0.82, 5.05) | 0.126 |  | 9 | 365 395 | 1.55 (0.55, 4.36) | 0.409 |  | 31 | 365 549 | 1.51 (0.87, 2.63) | 0.146 |
| Q4 | 5 | 364 851 | 0.72 (0.23, 2.27) | 0.572 |  | 9 | 364 809 | 1.55 (0.55, 4.38) | 0.408 |  | 35 | 364 958 | 1.70 (0.98, 2.92) | 0.057 |
| Q5 | 16 | 363 710 | 2.10 (0.85, 5.16) | 0.107 |  | 5 | 363 720 | 0.85 (0.26, 2.82) | 0.792 |  | 50 | 363 784 | 2.33 (1.39, 3.91) | **0.001** |
| P-trend |  |  |  | 0.408 |  |  |  |  | 0.918 |  |  |  |  | **<0.001** |

HR was calculated by Cox model adjusted for age, age-squared, sex, ethnicity, TDI, smoking status, drinking status, education levels, physical activities, BMI, PRS, and total energy (if the exposure was energy or energy proportion, total energy intake will not be adjusted)

# Supplementary table 9. Subgroup analyses for the association between UPF intake and risk of CD and UC stratified by age

|  | **CD** | | |  | **UC** | | |
| --- | --- | --- | --- | --- | --- | --- | --- |
|  | **P for**  **interaction** | **Age<60**  **HR 95%CI** | **Age>60**  **HR 95%CI** |  | **P for**  **interaction** | **Age<60**  **HR 95%CI** | **Age>60**  **HR 95%CI** |
| Serving |  |  |  |  |  |  |  |
| Per SD | 0.706 | **1.18 (1.01, 1.38)** | 1.16 (0.95, 1.42) |  | 0.785 | 0.97 (0.86, 1.09) | 0.98 (0.86, 1.13) |
| Q1 | 0.436 | Ref | Ref |  | 0.504 | Ref | Ref |
| Q2 |  | 0.78 (0.43, 1.41) | 1.30 (0.62, 2.73) |  |  | 1.18 (0.83, 1.69) | 0.70 (0.44, 1.11) |
| Q3 |  | 1.17 (0.68, 2.01) | 0.99 (0.45, 2.18) |  |  | 1.32 (0.93, 1.88) | 1.15 (0.76, 1.74) |
| Q4 |  | **1.89 (1.13, 3.16)** | 1.56 (0.75, 3.25) |  |  | 0.98 (0.67, 1.43) | 0.88 (0.57, 1.36) |
| Q5 |  | 1.52 (0.86, 2.69) | 1.96 (0.92, 4.15) |  |  | 1.02 (0.69, 1.51) | 1.01 (0.64, 1.58) |
| P-trend |  | **0.009** | 0.059 |  |  | 0.656 | 0.661 |
| Energy |  |  |  |  |  |  |  |
| Per SD | 0.375 | 1.11 (0.95, 1.28) | **1.23 (1.02, 1.47)** |  | 0.834 | 1.06 (0.96, 1.17) | 1.07 (0.95, 1.21) |
| Q1 | 0.710 | Ref | Ref |  | 0.318 | Ref | Ref |
| Q2 |  | 1.03 (0.60, 1.76) | 0.83 (0.40, 1.72) |  |  | 0.91 (0.65, 1.28) | 0.84 (0.54, 1.31) |
| Q3 |  | 1.06 (0.62, 1.80) | 0.95 (0.47, 1.92) |  |  | 0.94 (0.67, 1.31) | 0.97 (0.64, 1.49) |
| Q4 |  | 1.26 (0.76, 2.11) | 1.61 (0.86, 3.02) |  |  | 0.84 (0.60, 1.18) | 1.50 (1.02, 2.21) |
| Q5 |  | 1.29 (0.78, 2.13) | 1.63 (0.87, 3.07) |  |  | 1.02 (0.73, 1.41) | 1.20 (0.80, 1.81) |
| P-trend |  | 0.216 | **0.022** |  |  | 0.933 | **0.039** |
| Energy% |  |  |  |  |  |  |  |
| Per SD | 0.911 | 1.24 (1.06, 1.44) | 1.21 (0.99, 1.48) |  | 0.903 | 1.00 (0.90, 1.11) | 1.01 (0.89, 1.15) |
| Q1 | 0.288 | Ref | Ref |  | 0.797 | Ref | Ref |
| Q2 |  | 1.06 (0.60, 1.88) | 1.74 (0.83, 3.66) |  |  | 0.85 (0.60, 1.20) | 0.92 (0.61, 1.39) |
| Q3 |  | 1.51 (0.89, 2.57) | 1.82 (0.87, 3.80) |  |  | 0.98 (0.71, 1.36) | 1.05 (0.70, 1.57) |
| Q4 |  | 1.10 (0.62, 1.94) | **2.16 (1.06, 4.43)** |  |  | 1.06 (0.77, 1.46) | 1.20 (0.81, 1.77) |
| Q5 |  | **1.93 (1.17, 3.20)** | 2.01 (0.97, 4.16) |  |  | 0.83 (0.59, 1.16) | 0.93 (0.62, 1.40) |
| P-trend |  | **0.011** | **0.057** |  |  | 0.66 | 0.781 |

HR was calculated by Cox model adjusted for sex, ethnicity, TDI, smoking status, drinking status, education levels, physical activities, BMI, PRS, and total energy (if the exposure was energy or energy proportion, total energy intake will not be adjusted)

# Supplementary table 10. Subgroup analyses for the association between UPF intake and risk of CD and UC stratified by sex

|  | **CD** | | |  | **UC** | | |
| --- | --- | --- | --- | --- | --- | --- | --- |
|  | **P for**  **interaction** | **Female**  **HR 95%CI** | **Male**  **HR 95%CI** |  | **P for**  **interaction** | **Female**  **HR 95%CI** | **Male**  **HR 95%CI** |
| Serving |  |  |  |  |  |  |  |
| Per SD | 0.070 | **1.23 (1.05, 1.45)** | 1.11 (0.93, 1.34) |  | 0.227 | 1.14 (0.91, 1.43) | 0.94 (0.82, 1.07) |
| Q1 | 0.080 | Ref | Ref |  | 0.739 | Ref | Ref |
| Q2 |  | 0.80 (0.41, 1.57) | 1.31 (0.75, 2.27) |  |  | 1.05 (0.69, 1.59) | 0.84 (0.58, 1.21) |
| Q3 |  | 0.79 (0.39, 1.60) | 0.87 (0.46, 1.65) |  |  | 1.38 (0.92, 2.08) | 0.95 (0.66, 1.37) |
| Q4 |  | 1.52 (0.83, 2.76) | 1.40 (0.78, 2.53) |  |  | 1.42 (0.95, 2.12) | 0.81 (0.55, 1.19) |
| Q5 |  | 1.71 (0.92, 3.17) | 1.62 (0.88, 2.99) |  |  | 1.24 (0.81, 1.89) | 0.83 (0.56, 1.24) |
| P-trend |  | **0.010** | 0.149 |  |  | 0.157 | 0.409 |
| Energy |  |  |  |  |  |  |  |
| Per SD | 0.038 | **1.27 (1.09, 1.48)** | 1.06 (0.89, 1.25) |  | 0.934 | 1.05 (0.94, 1.17) | 1.07 (0.96, 1.20) |
| Q1 | 0.275 | Ref | Ref |  | 0.771 | Ref | Ref |
| Q2 |  | 0.87 (0.45, 1.69) | 0.74 (0.41, 1.31) |  |  | 1.10 (0.75, 1.61) | 0.89 (0.62, 1.28) |
| Q3 |  | 1.31 (0.72, 2.40) | 1.12 (0.67, 1.88) |  |  | 1.14 (0.78, 1.66) | 0.98 (0.68, 1.40) |
| Q4 |  | 1.44 (0.80, 2.60) | 0.90 (0.52, 1.56) |  |  | 1.39 (0.96, 1.99) | 1.00 (0.70, 1.42) |
| Q5 |  | 1.69 (0.96, 2.98) | 1.09 (0.64, 1.83) |  |  | 1.09 (0.75, 1.60) | 1.11 (0.79, 1.58) |
| P-trend |  | **0.018** | 0.580 |  |  | 0.332 | 0.408 |
| Energy% |  |  |  |  |  |  |  |
| Per SD | 0.146 | **1.32 (1.11, 1.57)** | 1.16 (0.98, 1.38) |  | 0.913 | 1.00 (0.89, 1.12) | 1.01 (0.90, 1.13) |
| Q1 | 0.321 | Ref | Ref |  | 0.389 | Ref | Ref |
| Q2 |  | 1.31 (0.65, 2.64) | 0.99 (0.55, 1.78) |  |  | 1.00 (0.67, 1.47) | 0.77 (0.54, 1.11) |
| Q3 |  | **1.96 (1.03, 3.74)** | 1.51 (0.89, 2.58) |  |  | 1.30 (0.90, 1.87) | 0.83 (0.58, 1.19) |
| Q4 |  | 1.42 (0.72, 2.82) | 1.02 (0.57, 1.84) |  |  | 1.36 (0.94, 1.95) | 1.04 (0.74, 1.46) |
| Q5 |  | **2.66 (1.44, 4.92)** | 1.44 (0.84, 2.49) |  |  | 0.91 (0.62, 1.35) | 0.87 (0.61, 1.24) |
| P-trend |  | **0.002** | 0.215 |  |  | 0.740 | 0.973 |

HR was calculated by Cox model adjusted for age, age-squared, ethnicity, TDI, smoking status, drinking status, education levels, physical activities, BMI, PRS, and total energy (if the exposure was energy or energy proportion, total energy intake will not be adjusted)

# Supplementary table 11. Subgroup analyses for the association between UPF intake and risk of CD and UC stratified by alcohol drinking status

|  | **CD** | | | |  | **UC** | | | |
| --- | --- | --- | --- | --- | --- | --- | --- | --- | --- |
|  | **P for interaction** | **Current drinking**  **HR 95%CI** | **Previous drinking**  **HR 95%CI** | **Never drinking**  **HR 95%CI** |  | **P for interaction** | **Current drinking**  **HR 95%CI** | **Previous drinking**  **HR 95%CI** | **Never drinking**  **HR 95%CI** |
| Serving |  |  |  |  |  |  |  |  |  |
| Per SD | 0.188 | 1.13 (0.99, 1.30) | **1.96 (1.25, 3.08)** | 1.04 (0.57, 1.92) |  | 0.653 | 0.97 (0.88, 1.06) | 0.91 (0.55, 1.48) | 1.25 (0.87, 1.80) |
| Q1 | **0.045** | Ref | Ref | Ref |  | 0.807 | Ref | Ref | Ref |
| Q2 |  | 0.88 (0.55, 1.41) | - | 2.57 (0.44, 14.93) |  |  | 0.98 (0.73, 1.32) | 0.76 (0.24, 2.42) | 1.29 (0.28, 5.96) |
| Q3 |  | 1.07 (0.68, 1.70) | - | 0.71 (0.06, 8.54) |  |  | 1.27 (0.96, 1.68) | 0.36 (0.09, 1.51) | 2.47 (0.59, 10.35) |
| Q4 |  | **1.61 (1.04, 2.50)** | - | 2.82 (0.39, 20.71) |  |  | 1.02 (0.76, 1.38) | 0.58 (0.17, 2.00) | 1.14 (0.21, 6.33) |
| Q5 |  | 1.41 (0.88, 2.25) | - | 2.62 (0.33, 20.84) |  |  | 0.99 (0.73, 1.35) | 0.25 (0.05, 1.23) | 2.89 (0.62, 13.37) |
| P-trend |  | **0.015** | - | 0.447 |  |  | 0.986 | 0.096 | 0.219 |
| Energy |  |  |  |  |  |  |  |  |  |
| Per SD | 0.079 | 1.12 (0.99, 1.26) | **2.16 (1.38, 3.38)** | 1.03 (0.59, 1.81) |  | 0.567 | 1.07 (0.98, 1.16) | 0.85 (0.55, 1.32) | 1.20 (0.82, 1.75) |
| Q1 | **0.034** | Ref | Ref | Ref |  | 0.230 | Ref | Ref | Ref |
| Q2 |  | 0.91 (0.58, 1.42) | - | 2.06 (0.37, 11.29) |  |  | 0.87 (0.65, 1.15) | 0.64 (0.20, 2.04) | 0.56 (0.16, 1.94) |
| Q3 |  | 0.89 (0.57, 1.40) | - | 1.64 (0.27, 10.04) |  |  | 1.08 (0.83, 1.41) | 0.61 (0.19, 1.96) | 0.28 (0.06, 1.36) |
| Q4 |  | 1.33 (0.88, 1.99) | - | 1.15 (0.16, 8.42) |  |  | 1.12 (0.85, 1.46) | 0.38 (0.10, 1.48) | 0.56 (0.16, 1.95) |
| Q5 |  | 1.24 (0.82, 1.88) | - | 1.18 (0.16, 8.69) |  |  | 1.12 (0.86, 1.47) | 0.47 (0.13, 1.68) | 1.22 (0.43, 3.47) |
| P-trend |  | 0.083 | - | 0.873 |  |  | 0.127 | 0.165 | 0.709 |
| Energy% |  |  |  |  |  |  |  |  |  |
| Per SD | **0.044** | **1.18 (1.04, 1.35)** | **2.81 (1.54, 5.13)** | 1.27 (0.75, 2.16) |  | 0.292 | 1.02 (0.93, 1.11) | 0.72 (0.48, 1.09) | 1.15 (0.77, 1.71) |
| Q1 | **0.047** | Ref | Ref | Ref |  | 0.153 | Ref | Ref | Ref |
| Q2 |  | 1.15 (0.73, 1.82) | - | - |  |  | 0.84 (0.64, 1.11) | 0.55 (0.18, 1.70) | 1.90 (0.57, 6.38) |
| Q3 |  | 1.41 (0.91, 2.19) | - | - |  |  | 1.00 (0.76, 1.30) | 0.54 (0.18, 1.68) | 0.73 (0.16, 3.31) |
| Q4 |  | 1.20 (0.76, 1.90) | - | - |  |  | 1.09 (0.84, 1.42) | 0.11 (0.01, 0.85) | 0.94 (0.23, 3.82) |
| Q5 |  | **1.81 (1.19, 2.76)** | - | - |  |  | 0.95 (0.72, 1.24) | 0.52 (0.17, 1.64) | 1.46 (0.40, 5.30) |
| P-trend |  | **0.008** | - | - |  |  | 0.646 | 0.094 | 0.956 |

HR was calculated by Cox model adjusted for age, age-squared, sex, ethnicity, TDI, smoking status, education levels, physical activities, BMI, PRS, and total energy (if the exposure was energy or energy proportion, total energy intake will not be adjusted)

# Supplementary table 12. Subgroup analyses for the association between UPF intake and risk of CD and UC stratified by smoking status

|  | **CD** | | | |  | **UC** | | | |
| --- | --- | --- | --- | --- | --- | --- | --- | --- | --- |
|  | **P for interaction** | **Current smoking**  **HR 95%CI** | **Previous smoking**  **HR 95%CI** | **Never smoking**  **HR 95%CI** |  | **P for interaction** | **Current smoking**  **HR 95%CI** | **Previous smoking**  **HR 95%CI** | **Never smoking**  **HR 95%CI** |
| Serving |  |  |  |  |  |  |  |  |  |
| Per SD | 0.851 | 1.22 (0.84, 1.78) | 1.15 (0.94, 1.42) | 1.18 (0.99, 1.40) |  | 0.488 | 0.94 (0.70, 1.27) | 0.92 (0.80, 1.06) | 1.05 (0.92, 1.19) |
| Q1 | 0.821 | Ref | Ref | Ref |  | 0.670 | Ref | Ref | Ref |
| Q2 |  | 2.31 (0.59, 9.10) | 1.28 (0.65, 2.54) | 0.60 (0.30, 1.19) |  |  | 1.23 (0.48, 3.13) | 0.70 (0.46, 1.08) | 1.22 (0.81, 1.82) |
| Q3 |  | 0.92 (0.18, 4.71) | 0.79 (0.35, 1.74) | 1.11 (0.61, 2.03) |  |  | 2.20 (0.95, 5.10) | 1.21 (0.82, 1.77) | 1.16 (0.76, 1.76) |
| Q4 |  | 2.11 (0.51, 8.82) | 1.33 (0.66, 2.71) | **1.98 (1.14, 3.43)** |  |  | 1.13 (0.43, 2.94) | 0.79 (0.52, 1.19) | 1.04 (0.67, 1.59) |
| Q5 |  | 1.92 (0.42, 8.82) | 1.74 (0.85, 3.56) | 1.47 (0.80, 2.69) |  |  | 1.04 (0.37, 2.87) | 0.77 (0.49, 1.19) | 1.26 (0.82, 1.93) |
| P-trend |  | 0.552 | 0.146 | **0.007** |  |  | 0.922 | 0.376 | 0.562 |
| Energy |  |  |  |  |  |  |  |  |  |
| Per SD | 0.397 | 1.27 (0.91, 1.77) | 1.05 (0.85, 1.28) | **1.21 (1.04, 1.41)** |  | 0.626 | 1.01 (0.78, 1.30) | 1.04 (0.92, 1.17) | 1.10 (0.98, 1.24) |
| Q1 | 0.111 | Ref | Ref | Ref |  | 0.097 | Ref | Ref | Ref |
| Q2 |  | 5.33 (0.64, 44.41) | 0.95 (0.50, 1.82) | 0.95 (0.51, 1.77) |  |  | 1.60 (0.66, 3.86) | 1.00 (0.66, 1.52) | 0.76 (0.52, 1.12) |
| Q3 |  | 4.34 (0.51, 37.23) | 0.68 (0.33, 1.38) | 1.22 (0.68, 2.20) |  |  | 2.08 (0.90, 4.84) | 1.25 (0.84, 1.86) | 0.67 (0.45, 1.00) |
| Q4 |  | 4.77 (0.57, 39.96) | 1.02 (0.54, 1.93) | 1.66 (0.95, 2.89) |  |  | 1.31 (0.52, 3.28) | 1.31 (0.89, 1.94) | 0.94 (0.65, 1.35) |
| Q5 |  | 5.64 (0.70, 45.70) | 1.03 (0.55, 1.95) | **1.82 (1.05, 3.14)** |  |  | 1.34 (0.53, 3.39) | 1.15 (0.77, 1.72) | 1.07 (0.75, 1.53) |
| P-trend |  | 0.189 | 0.844 | **0.005** |  |  | 0.778 | 0.244 | 0.399 |
| Energy% |  |  |  |  |  |  |  |  |  |
| Per SD | 0.117 | 1.61 (1.10, 2.35) | 1.06 (0.86, 1.30) | **1.32 (1.12, 1.56)** |  | 0.849 | 1.00 (0.77, 1.30) | 0.98 (0.86, 1.10) | 1.04 (0.92, 1.17) |
| Q1 | 0.511 | Ref | Ref | Ref |  | 0.502 | Ref | Ref | Ref |
| Q2 |  | 0.47 (0.04, 5.21) | 1.45 (0.76, 2.74) | 1.29 (0.67, 2.49) |  |  | 1.70 (0.71, 4.07) | 1.07 (0.71, 1.59) | 0.65 (0.43, 0.97) |
| Q3 |  | 2.80 (0.56, 13.93) | 0.82 (0.39, 1.70) | **2.36 (1.30, 4.26)** |  |  | 2.05 (0.89, 4.77) | 1.06 (0.71, 1.58) | 0.93 (0.64, 1.34) |
| Q4 |  | 3.22 (0.66, 15.55) | 0.98 (0.49, 1.97) | 1.64 (0.87, 3.08) |  |  | 1.63 (0.68, 3.90) | 1.38 (0.95, 2.01) | 0.86 (0.59, 1.25) |
| Q5 |  | 4.40 (0.95, 20.36) | 1.35 (0.71, 2.59) | **2.33 (1.29, 4.23)** |  |  | 0.80 (0.29, 2.23) | 0.90 (0.59, 1.36) | 0.97 (0.67, 1.39) |
| P-trend |  | **0.007** | 0.758 | **0.005** |  |  | 0.692 | 0.852 | 0.698 |

HR was calculated by Cox model adjusted for age, age-squared, sex, ethnicity, TDI, drinking status, education levels, physical activities, BMI, PRS, and total energy (if the exposure was energy or energy proportion, total energy intake will not be adjusted)

# Supplementary table 13. Subgroup analyses for the association between UPF intake and risk of CD and UC stratified by BMI

|  | **CD** | | |  | **UC** | | |
| --- | --- | --- | --- | --- | --- | --- | --- |
|  | **P for**  **interaction** | **BMI<=30**  **HR 95%CI** | **BMI>30**  **HR 95%CI** |  | **P for**  **interaction** | **BMI<=30**  **HR 95%CI** | **BMI>30**  **HR 95%CI** |
| Serving |  |  |  |  |  |  |  |
| Per SD | 0.986 | 1.14 (0.98, 1.32) | 1.23 (0.97, 1.55) |  | 0.179 | 1.04 (0.94, 1.15) | 0.82 (0.68, 0.99) |
| Q1 | 0.440 | Ref | Ref |  | 0.320 | Ref | Ref |
| Q2 |  | 0.90 (0.54, 1.49) | 1.26 (0.55, 2.88) |  |  | 0.90 (0.66, 1.25) | 1.14 (0.66, 1.98) |
| Q3 |  | 0.87 (0.51, 1.48) | 0.93 (0.39, 2.24) |  |  | 1.20 (0.88, 1.64) | 1.38 (0.83, 2.30) |
| Q4 |  | 1.28 (0.79, 2.07) | **2.38 (1.10, 5.15)** |  |  | 0.97 (0.70, 1.33) | 0.77 (0.43, 1.40) |
| Q5 |  | 1.39 (0.84, 2.31) | 1.59 (0.68, 3.73) |  |  | 1.16 (0.84, 1.62) | 0.70 (0.38, 1.28) |
| P-trend |  | 0.075 | 0.092 |  |  | 0.334 | 0.086 |
| Energy |  |  |  |  |  |  |  |
| Per SD | 0.612 | **1.15 (1.01, 1.32)** | 1.15 (0.92, 1.44) |  | 0.848 | 1.06 (0.97, 1.17) | 1.06 (0.90, 1.24) |
| Q1 | 0.790 | Ref | Ref |  | 0.390 | Ref | Ref |
| Q2 |  | 0.89 (0.54, 1.46) | 1.24 (0.54, 2.88) |  |  | 0.97 (0.71, 1.34) | 0.70 (0.43, 1.17) |
| Q3 |  | 0.84 (0.51, 1.40) | 1.68 (0.76, 3.71) |  |  | 1.11 (0.82, 1.51) | 0.80 (0.49, 1.31) |
| Q4 |  | 1.23 (0.77, 1.95) | 1.79 (0.82, 3.94) |  |  | 1.23 (0.91, 1.66) | 0.66 (0.39, 1.10) |
| Q5 |  | 1.31 (0.83, 2.07) | 1.69 (0.76, 3.76) |  |  | 1.23 (0.91, 1.67) | 0.86 (0.53, 1.38) |
| P-trend |  | 0.086 | 0.122 |  |  | 0.061 | 0.487 |
| Energy% |  |  |  |  |  |  |  |
| Per SD | 0.908 | **1.21 (1.05, 1.40)** | **1.27 (1.01, 1.60)** |  | 0.218 | 1.04 (0.94, 1.14) | 0.92 (0.78, 1.08) |
| Q1 | 0.959 | Ref | Ref |  | 0.082 | Ref | Ref |
| Q2 |  | 1.01 (0.60, 1.71) | 2.56 (0.99, 6.60) |  |  | 0.96 (0.70, 1.32) | 0.65 (0.40, 1.07) |
| Q3 |  | 1.47 (0.91, 2.38) | **2.75 (1.08, 7.05)** |  |  | 1.17 (0.87, 1.58) | 0.72 (0.45, 1.17) |
| Q4 |  | 1.20 (0.73, 1.98) | **2.60 (1.00, 6.71)** |  |  | 1.26 (0.94, 1.69) | 0.71 (0.44, 1.15) |
| Q5 |  | **1.67 (1.04, 2.68)** | **3.14 (1.25, 7.93)** |  |  | 1.08 (0.79, 1.46) | 0.64 (0.39, 1.06) |
| P-trend |  | **0.024** | **0.034** |  |  | **0.231** | 0.137 |

HR was calculated by Cox model adjusted for age, age-squared, sex, ethnicity, TDI, smoking status, drinking status, education levels, physical activities, PRS, and total energy (if the exposure was energy or energy proportion, total energy intake will not be adjusted)

# Supplementary table 14. Subgroup analyses for the association between UPF intake and risk of CD and UC stratified by PRS

|  | **CD** | | | |  | **UC** | | | |
| --- | --- | --- | --- | --- | --- | --- | --- | --- | --- |
|  | **P for interaction** | **Low**  **HR 95%CI** | **Intermediate**  **HR 95%CI** | **High**  **HR 95%CI** |  | **P for interaction** | **Low**  **HR 95%CI** | **Intermediate**  **HR 95%CI** | **High**  **HR 95%CI** |
| Serving |  |  |  |  |  |  |  |  |  |
| Per SD | 0.894 | 1.01 (0.69, 1.47) | **1.22 (1.03, 1.43)** | 1.14 (0.91, 1.43) |  | 0.931 | 1.00 (0.78, 1.28) | 0.98 (0.87, 1.11) | 0.96 (0.82, 1.13) |
| Q1 | 0.508 | Ref | Ref | Ref |  | 0.942 | Ref | Ref | Ref |
| Q2 |  | 0.72 (0.23, 2.32) | 0.69 (0.37, 1.30) | 1.66 (0.72, 3.83) |  |  | 0.55 (0.26, 1.16) | 1.14 (0.78, 1.66) | 1.01 (0.61, 1.68) |
| Q3 |  | 0.77 (0.25, 2.39) | 1.00 (0.55, 1.80) | 1.51 (0.63, 3.60) |  |  | 0.89 (0.46, 1.70) | 1.29 (0.89, 1.88) | 1.52 (0.95, 2.44) |
| Q4 |  | 0.71 (0.22, 2.27) | 1.66 (0.96, 2.89) | **2.87 (1.27, 6.46)** |  |  | 0.46 (0.21, 1.00) | 1.25 (0.85, 1.83) | 0.86 (0.50, 1.47) |
| Q5 |  | 0.75 (0.23, 2.46) | 1.62 (0.90, 2.89) | 2.30 (0.97, 5.49) |  |  | 0.59 (0.27, 1.26) | 1.07 (0.71, 1.61) | 1.19 (0.70, 2.01) |
| P-trend |  | 0.674 | **0.006** | **0.023** |  |  | 0.166 | 0.725 | 0.784 |
| Energy |  |  |  |  |  |  |  |  |  |
| Per SD | 0.635 | 1.30 (0.96, 1.74) | 1.15 (0.99, 1.35) | 1.10 (0.89, 1.36) |  | 0.514 | 0.94 (0.75, 1.18) | 1.06 (0.95, 1.18) | 1.12 (0.98, 1.29) |
| Q1 | 0.768 | Ref | Ref | Ref |  | 0.662 | Ref | Ref | Ref |
| Q2 |  | 0.85 (0.23, 3.17) | 0.94 (0.53, 1.66) | 1.01 (0.47, 2.19) |  |  | 0.63 (0.31, 1.31) | 0.84 (0.58, 1.21) | 1.11 (0.69, 1.80) |
| Q3 |  | 1.07 (0.31, 3.74) | 0.89 (0.50, 1.58) | 1.08 (0.51, 2.31) |  |  | 0.87 (0.45, 1.69) | 1.05 (0.75, 1.49) | 1.01 (0.62, 1.65) |
| Q4 |  | 1.71 (0.55, 5.31) | 1.16 (0.68, 1.99) | 1.76 (0.89, 3.51) |  |  | 0.85 (0.44, 1.64) | 1.11 (0.79, 1.56) | 1.21 (0.75, 1.94) |
| Q5 |  | 1.96 (0.65, 5.87) | 1.51 (0.91, 2.53) | 1.24 (0.59, 2.60) |  |  | 0.61 (0.30, 1.26) | 1.05 (0.74, 1.48) | 1.43 (0.91, 2.27) |
| P-trend |  | 0.103 | 0.064 | 0.218 |  |  | 0.379 | 0.368 | 0.114 |
| Energy% |  |  |  |  |  |  |  |  |  |
| Per SD | 0.661 | **1.40 (1.00, 1.96)** | **1.21 (1.03, 1.43)** | 1.22 (0.98, 1.51) |  | 0.278 | 0.84 (0.67, 1.05) | 1.01 (0.91, 1.13) | 1.08 (0.93, 1.25) |
| Q1 | 0.635 | Ref | Ref | Ref |  | 0.488 | Ref | Ref | Ref |
| Q2 |  | **4.76 (1.03, 22.11)** | 1.12 (0.61, 2.06) | 1.00 (0.45, 2.22) |  |  | 0.90 (0.46, 1.76) | 0.81 (0.56, 1.15) | 0.86 (0.52, 1.41) |
| Q3 |  | 3.17 (0.64, 15.80) | 1.59 (0.90, 2.79) | 1.57 (0.76, 3.25) |  |  | 0.88 (0.45, 1.74) | 0.92 (0.65, 1.30) | 1.31 (0.84, 2.05) |
| Q4 |  | 2.59 (0.50, 13.45) | 1.49 (0.84, 2.64) | 1.05 (0.48, 2.32) |  |  | 1.06 (0.56, 2.02) | 0.98 (0.70, 1.37) | 1.21 (0.77, 1.92) |
| Q5 |  | **4.82 (1.04, 22.28)** | **1.85 (1.07, 3.22)** | 1.82 (0.90, 3.67) |  |  | 0.39 (0.17, 0.90) | 0.96 (0.69, 1.35) | 1.09 (0.68, 1.74) |
| P-trend |  | 0.172 | **0.016** | 0.102 |  |  | 0.105 | 0.798 | 0.363 |

HR was calculated by Cox model adjusted for age, age-squared, sex, ethnicity, TDI, smoking status, drinking status, education levels, physical activities, BMI, and total energy (if the exposure was energy or energy proportion, total energy intake will not be adjusted)

# Supplementary table 15. Sensitivity analyses for the association between UPF intake and risk of CD and UC when excluding liquid UPF food

|  | **CD** | |  | **UC** | |
| --- | --- | --- | --- | --- | --- |
|  | **HR 95%CI** | **P** |  | **HR 95%CI** | **P** |
| Serving |  |  |  |  |  |
| Per SD | 1.20 (1.07, 1.35) | **0.002** |  | 1.01 (0.92, 1.10) | 0.892 |
| Q1 | Ref |  |  | Ref |  |
| Q2 | 1.12 (0.69, 1.80) | 0.656 |  | 0.99 (0.75, 1.32) | 0.965 |
| Q3 | 1.26 (0.81, 1.98) | 0.308 |  | 1.07 (0.82, 1.39) | 0.638 |
| Q4 | 2.12 (1.37, 3.29) | **0.001** |  | 0.99 (0.74, 1.33) | 0.966 |
| Q5 | 2.03 (1.28, 3.21) | **0.002** |  | 1.08 (0.81, 1.44) | 0.603 |
| P-trend |  | **<0.001** |  |  | 0.639 |
| Energy |  |  |  |  |  |
| Per SD | 1.17 (1.04, 1.31) | **0.007** |  | 1.06 (0.98, 1.14) | 0.174 |
| Q1 | Ref |  |  | Ref |  |
| Q2 | 0.93 (0.60, 1.44) | 0.732 |  | 0.96 (0.74, 1.25) | 0.760 |
| Q3 | 1.07 (0.70, 1.64) | 0.751 |  | 1.00 (0.77, 1.30) | 0.996 |
| Q4 | 1.37 (0.92, 2.05) | 0.124 |  | 1.02 (0.78, 1.32) | 0.895 |
| Q5 | 1.60 (1.08, 2.37) | **0.018** |  | 1.15 (0.89, 1.48) | 0.286 |
| P-trend |  | **0.003** |  |  | 0.241 |
| Energy% |  |  |  |  |  |
| Per SD | 1.25 (1.11, 1.42) | **<0.001** |  | 1.00 (0.93, 1.09) | 0.926 |
| Q1 | Ref |  |  | Ref |  |
| Q2 | 1.49 (0.94, 2.35) | 0.091 |  | 0.94 (0.72, 1.22) | 0.651 |
| Q3 | 1.65 (1.05, 2.58) | **0.030** |  | 0.96 (0.74, 1.25) | 0.760 |
| Q4 | 1.94 (1.25, 3.01) | **0.003** |  | 1.13 (0.88, 1.45) | 0.357 |
| Q5 | 2.07 (1.35, 3.19) | **0.001** |  | 0.97 (0.75, 1.26) | 0.830 |
| P-trend |  | **<0.001** |  |  | 0.675 |

HR was calculated by Cox model adjusted for age, age-squared, sex, ethnicity, TDI, smoking status, drinking status, education levels, physical activities, BMI, PRS, and total energy (if the exposure was energy or energy proportion, total energy intake will not be adjusted).

# Supplementary table 16. Sensitivity analyses for the association between UPF intake and risk of CD and UC when further adjusting for nutrients intake and AHEI

|  | **Further adjusted for total fat, total carbohydrate, total protein intake** | |  | **Further adjusted for total sugar and total fiber intake** | | **Further adjusted for saturated fat**  **and polyunsaturated fat intake** |  | **Further adjusted for AHEI** |  |
| --- | --- | --- | --- | --- | --- | --- | --- | --- | --- |
|  | **HR 95%CI** | **P** |  | **HR 95%CI** | **P** | **HR 95%CI** | **P** | **HR 95%CI** | **P** |
| **CD** |  |  |  |  |  |  |  |  |  |
| Serving |  |  |  |  |  |  |  |  |  |
| Per SD | 1.13 (0.99, 1.29) | 0.065 |  | 1.17 (1.03, 1.32) | **0.016** | 1.15 (1.01, 1.31) | **0.029** | 1.16 (1.03, 1.32) | **0.018** |
| Q1 | Ref |  |  | Ref |  | Ref |  | Ref |  |
| Q2 | 0.89 (0.57, 1.40) | 0.623 |  | 0.92 (0.58, 1.44) | 0.703 | 0.91 (0.58, 1.43) | 0.680 | 0.92 (0.58, 1.44) | 0.703 |
| Q3 | 1.04 (0.66, 1.63) | 0.857 |  | 1.09 (0.70, 1.70) | 0.713 | 1.07 (0.69, 1.68) | 0.759 | 1.09 (0.69, 1.70) | 0.716 |
| Q4 | 1.63 (1.07, 2.50) | **0.024** |  | 1.74 (1.14, 2.65) | **0.010** | 1.70 (1.11, 2.60) | **0.014** | 1.74 (1.14, 2.65) | **0.011** |
| Q5 | 1.47 (0.93, 2.32) | 0.102 |  | 1.61 (1.03, 2.51) | **0.037** | 1.56 (0.99, 2.44) | 0.055 | 1.61 (1.02, 2.52) | **0.039** |
| P-trend |  | **0.008** |  |  | **0.002** |  | **0.003** |  | **0.002** |
| Energy |  |  |  |  |  |  |  |  |  |
| Per SD | 1.31 (1.08, 1.58) | **0.006** |  | 1.21 (1.06, 1.37) | **0.004** | 1.22 (1.05, 1.42) | **0.009** | 1.16 (1.03, 1.31) | **0.017** |
| Q1 | Ref |  |  | Ref |  | Ref |  | Ref |  |
| Q2 | 1.04 (0.67, 1.61) | 0.862 |  | 1.00 (0.65, 1.54) | 0.989 | 1.00 (0.65, 1.55) | 0.986 | 0.98 (0.64, 1.51) | 0.929 |
| Q3 | 1.05 (0.66, 1.65) | 0.848 |  | 0.99 (0.64, 1.53) | 0.950 | 0.99 (0.64, 1.55) | 0.975 | 0.95 (0.62, 1.48) | 0.830 |
| Q4 | 1.65 (1.05, 2.59) | **0.031** |  | 1.51 (1.01, 2.27) | **0.044** | 1.53 (1.00, 2.34) | **0.048** | 1.44 (0.96, 2.15) | 0.079 |
| Q5 | 1.87 (1.09, 3.21) | **0.023** |  | 1.63 (1.07, 2.49) | **0.023** | 1.66 (1.04, 2.65) | **0.034** | 1.48 (0.98, 2.24) | 0.062 |
| P-trend |  | **0.006** |  |  | **0.004** |  | **0.008** |  | **0.013** |
| Energy% |  |  |  |  |  |  |  |  |  |
| Per SD | 1.25 (1.09, 1.43) | **0.001** |  | 1.24 (1.10, 1.40) | **0.001** | 1.24 (1.09, 1.40) | **0.001** | 1.24 (1.09, 1.40) | **0.001** |
| Q1 | Ref |  |  | Ref |  | Ref |  | Ref |  |
| Q2 | 1.29 (0.82, 2.04) | 0.265 |  | 1.29 (0.82, 2.02) | 0.269 | 1.29 (0.82, 2.02) | 0.276 | 1.29 (0.82, 2.02) | 0.276 |
| Q3 | 1.63 (1.05, 2.54) | **0.028** |  | 1.62 (1.05, 2.50) | **0.028** | 1.62 (1.05, 2.49) | **0.030** | 1.62 (1.05, 2.49) | **0.030** |
| Q4 | 1.48 (0.94, 2.33) | 0.092 |  | 1.46 (0.94, 2.27) | 0.090 | 1.46 (0.94, 2.28) | 0.096 | 1.46 (0.93, 2.27) | 0.097 |
| Q5 | 2.05 (1.31, 3.22) | **0.002** |  | 2.02 (1.33, 3.07) | **0.001** | 2.01 (1.32, 3.07) | **0.001** | 2.01 (1.32, 3.06) | **0.001** |
| P-trend |  | **0.002** |  |  | **0.001** |  | **0.001** |  | **0.001** |
| **UC** |  |  |  |  |  |  |  |  |  |
| Serving |  |  |  |  |  |  |  |  |  |
| Per SD | 0.98 (0.90, 1.08) | 0.742 |  | 0.98 (0.90, 1.07) | 0.646 | 0.97 (0.89, 1.07) | 0.557 | 0.98 (0.90, 1.07) | 0.673 |
| Q1 | Ref |  |  | Ref |  | Ref |  | Ref |  |
| Q2 | 1.00 (0.76, 1.32) | 0.995 |  | 1.00 (0.75, 1.31) | 0.972 | 0.99 (0.75, 1.31) | 0.939 | 1.00 (0.76, 1.32) | 0.985 |
| Q3 | 1.27 (0.97, 1.67) | 0.080 |  | 1.26 (0.97, 1.65) | 0.089 | 1.25 (0.95, 1.64) | 0.104 | 1.27 (0.97, 1.66) | 0.085 |
| Q4 | 1.01 (0.75, 1.35) | 0.957 |  | 0.99 (0.75, 1.33) | 0.971 | 0.98 (0.73, 1.31) | 0.897 | 1.00 (0.75, 1.34) | 0.996 |
| Q5 | 1.03 (0.76, 1.40) | 0.846 |  | 1.01 (0.75, 1.36) | 0.949 | 0.99 (0.73, 1.34) | 0.954 | 1.02 (0.76, 1.37) | 0.909 |
| P-trend |  | 0.905 |  |  | 0.978 |  | 0.879 |  | 0.981 |
| Energy |  |  |  |  |  |  |  |  |  |
| Per SD | 1.06 (0.93, 1.19) | 0.387 |  | 1.04 (0.95, 1.13) | 0.397 | 1.02 (0.92, 1.13) | 0.682 | 1.07 (0.98, 1.16) | 0.124 |
| Q1 | Ref |  |  | Ref |  | Ref |  | Ref |  |
| Q2 | 0.87 (0.66, 1.14) | 0.318 |  | 0.87 (0.66, 1.14) | 0.301 | 0.86 (0.65, 1.12) | 0.266 | 0.88 (0.67, 1.16) | 0.367 |
| Q3 | 0.98 (0.75, 1.30) | 0.909 |  | 0.98 (0.76, 1.28) | 0.897 | 0.96 (0.74, 1.25) | 0.766 | 1.01 (0.78, 1.32) | 0.926 |
| Q4 | 1.05 (0.78, 1.40) | 0.763 |  | 1.05 (0.81, 1.36) | 0.732 | 1.01 (0.77, 1.33) | 0.942 | 1.10 (0.84, 1.42) | 0.492 |
| Q5 | 0.99 (0.70, 1.41) | 0.977 |  | 1.00 (0.76, 1.31) | 0.988 | 0.94 (0.69, 1.28) | 0.706 | 1.08 (0.83, 1.42) | 0.559 |
| P-trend |  | 0.633 |  |  | 0.567 |  | 0.883 |  | 0.237 |
| Energy% |  |  |  |  |  |  |  |  |  |
| Per SD | 1.02 (0.93, 1.11) | 0.713 |  | 1.00 (0.92, 1.08) | 0.996 | 0.99 (0.91, 1.07) | 0.767 | 1.00 (0.92, 1.09) | 0.919 |
| Q1 | Ref |  |  | Ref |  | Ref |  | Ref |  |
| Q2 | 0.83 (0.63, 1.08) | 0.164 |  | 0.82 (0.63, 1.07) | 0.151 | 0.81 (0.62, 1.06) | 0.126 | 0.83 (0.63, 1.08) | 0.161 |
| Q3 | 1.03 (0.79, 1.33) | 0.845 |  | 1.01 (0.79, 1.31) | 0.908 | 0.99 (0.77, 1.28) | 0.957 | 1.02 (0.79, 1.32) | 0.869 |
| Q4 | 1.04 (0.80, 1.35) | 0.787 |  | 1.02 (0.79, 1.31) | 0.889 | 0.99 (0.77, 1.27) | 0.931 | 1.03 (0.80, 1.32) | 0.835 |
| Q5 | 0.93 (0.70, 1.23) | 0.610 |  | 0.89 (0.69, 1.16) | 0.399 | 0.86 (0.66, 1.12) | 0.268 | 0.91 (0.70, 1.18) | 0.456 |
| P-trend |  | 0.807 |  |  | 0.944 |  | 0.714 |  | 0.980 |

HR was calculated by Cox model adjusted for age, age-squared, sex, ethnicity, TDI, smoking status, drinking status, education levels, physical activities, BMI, PRS, and total energy (if the exposure was energy or energy proportion, total energy intake will not be adjusted), nutrients intake or AHEI

# Supplementary table 17. Sensitivity analyses for the association between UPF intake and risk of CD and UC when further adjusting for urine sodium

|  | **CD** | |  | **UC** | |
| --- | --- | --- | --- | --- | --- |
|  | **HR 95%CI** | **P** |  | **HR 95%CI** | **P** |
| Serving |  |  |  |  |  |
| Per SD | 1.16 (1.02, 1.32) | **0.019** |  | 0.98 (0.89, 1.07) | 0.642 |
| Q1 | ref |  |  | ref |  |
| Q2 | 0.91 (0.58, 1.43) | 0.691 |  | 0.99 (0.75, 1.31) | 0.964 |
| Q3 | 1.08 (0.69, 1.69) | 0.736 |  | 1.26 (0.96, 1.65) | 0.092 |
| Q4 | 1.72 (1.13, 2.62) | **0.012** |  | 0.99 (0.74, 1.32) | 0.960 |
| Q5 | 1.58 (1.01, 2.47) | **0.044** |  | 1.01 (0.75, 1.36) | 0.955 |
| P-trend |  | **0.002** |  |  | 0.972 |
| Energy |  |  |  |  |  |
| Per SD | 1.15 (1.02, 1.29) | **0.019** |  | 1.06 (0.98, 1.15) | 0.122 |
| Q1 | ref |  |  | ref |  |
| Q2 | 0.97 (0.63, 1.50) | 0.903 |  | 0.88 (0.67, 1.15) | 0.359 |
| Q3 | 0.94 (0.61, 1.45) | 0.779 |  | 1.01 (0.78, 1.31) | 0.941 |
| Q4 | 1.41 (0.95, 2.09) | 0.091 |  | 1.09 (0.85, 1.41) | 0.500 |
| Q5 | 1.43 (0.97, 2.12) | 0.074 |  | 1.08 (0.83, 1.39) | 0.565 |
| P-trend |  | **0.014** |  |  | 0.227 |
| Energy% |  |  |  |  |  |
| Per SD | 1.23 (1.09, 1.39) | **0.001** |  | 1.01 (0.93, 1.09) | 0.870 |
| Q1 | ref |  |  | ref |  |
| Q2 | 1.28 (0.82, 2.01) | 0.283 |  | 0.83 (0.64, 1.08) | 0.166 |
| Q3 | 1.60 (1.04, 2.47) | **0.032** |  | 1.03 (0.80, 1.32) | 0.843 |
| Q4 | 1.44 (0.93, 2.23) | 0.106 |  | 1.03 (0.80, 1.33) | 0.799 |
| Q5 | 1.97 (1.30, 2.98) | **0.001** |  | 0.91 (0.70, 1.18) | 0.485 |
| P-trend |  | **0.002** |  |  | 0.932 |

HR was calculated by Cox model adjusted for age, age-squared, sex, ethnicity, TDI, smoking status, drinking status, education levels, physical activities, BMI, PRS, and total energy (if the exposure was energy or energy proportion, total energy intake will not be adjusted), urine sodium

# Supplementary table 18. Sensitivity analyses for the association between UPF intake and risk of CD and UC when further adjusting for Charlson Comorbidity Index

|  | **CD** | | **UC** | |
| --- | --- | --- | --- | --- |
|  | **HR 95%CI** | **P** |  |  |
| Serving |  |  |  |  |
| Per SD | 1.16 (1.03, 1.32) | **0.016** | 0.98 (0.89, 1.07) | 0.592 |
| Q1 | Ref |  | Ref |  |
| Q2 | 0.92 (0.58, 1.44) | 0.702 | 0.99 (0.75, 1.31) | 0.950 |
| Q3 | 1.09 (0.70, 1.70) | 0.713 | 1.26 (0.96, 1.64) | 0.096 |
| Q4 | 1.74 (1.14, 2.65) | **0.010** | 0.99 (0.74, 1.32) | 0.935 |
| Q5 | 1.60 (1.03, 2.50) | **0.038** | 1.00 (0.74, 1.34) | 0.994 |
| P-trend |  | **0.002** |  | 0.922 |
| Energy |  |  |  |  |
| Per SD | 1.15 (1.03, 1.30) | **0.014** | 1.06 (0.98, 1.15) | 0.130 |
| Q1 | Ref |  | Ref |  |
| Q2 | 0.98 (0.64, 1.50) | 0.911 | 0.88 (0.67, 1.15) | 0.357 |
| Q3 | 0.94 (0.61, 1.46) | 0.795 | 1.01 (0.78, 1.31) | 0.950 |
| Q4 | 1.42 (0.96, 2.11) | 0.082 | 1.09 (0.84, 1.41) | 0.508 |
| Q5 | 1.45 (0.98, 2.15) | 0.062 | 1.07 (0.83, 1.39) | 0.586 |
| P-trend |  | **0.011** |  | 0.238 |
| Energy% |  |  |  |  |
| Per SD | 1.24 (1.09, 1.40) | **0.001** | 1.00 (0.93, 1.09) | 0.908 |
| Q1 | Ref |  | Ref |  |
| Q2 | 1.28 (0.82, 2.01) | 0.276 | 0.83 (0.64, 1.08) | 0.165 |
| Q3 | 1.61 (1.05, 2.48) | **0.030** | 1.02 (0.80, 1.32) | 0.851 |
| Q4 | 1.45 (0.93, 2.25) | 0.097 | 1.03 (0.80, 1.32) | 0.819 |
| Q5 | 2.00 (1.32, 3.03) | **0.001** | 0.91 (0.70, 1.17) | 0.461 |
| P-trend |  | **0.001** |  | 0.967 |

HR was calculated by Cox model adjusted for age, age-squared, sex, ethnicity, TDI, smoking status, drinking status, education levels, physical activities, BMI, PRS, and total energy (if the exposure was energy or energy proportion, total energy intake will not be adjusted), Charlson Comorbidity Index (CCI).

# Supplementary table 19. Sensitivity analyses for the association between UPF intake and risk of CD and UC when further adjusting for C-reactive protein

|  | **CD** | |  | **UC** | |
| --- | --- | --- | --- | --- | --- |
|  | **HR 95%CI** | **P** |  | **HR 95%CI** | **P** |
| Serving |  |  |  |  |  |
| Per SD | 1.16 (1.03, 1.32) | **0.017** |  | 0.98 (0.89, 1.07) | 0.611 |
| Q1 | Ref |  |  | Ref |  |
| Q2 | 0.93 (0.59, 1.46) | 0.739 |  | 0.99 (0.75, 1.31) | 0.962 |
| Q3 | 1.09 (0.70, 1.71) | 0.696 |  | 1.26 (0.96, 1.64) | 0.095 |
| Q4 | 1.74 (1.14, 2.65) | **0.010** |  | 0.99 (0.74, 1.32) | 0.935 |
| Q5 | 1.60 (1.03, 2.50) | **0.038** |  | 1.00 (0.75, 1.35) | 0.990 |
| P-trend |  | **0.002** |  |  | 0.932 |
| Energy |  |  |  |  |  |
| Per SD | 1.16 (1.03, 1.30) | **0.014** |  | 1.06 (0.98, 1.15) | 0.130 |
| Q1 | Ref |  |  | Ref |  |
| Q2 | 0.98 (0.64, 1.51) | 0.942 |  | 0.88 (0.67, 1.15) | 0.358 |
| Q3 | 0.96 (0.62, 1.48) | 0.863 |  | 1.01 (0.78, 1.31) | 0.942 |
| Q4 | 1.44 (0.97, 2.13) | 0.073 |  | 1.09 (0.84, 1.41) | 0.510 |
| Q5 | 1.46 (0.99, 2.17) | 0.059 |  | 1.07 (0.83, 1.39) | 0.583 |
| P-trend |  | **0.010** |  |  | 0.239 |
| Energy% |  |  |  |  |  |
| Per SD | 1.23 (1.09, 1.39) | **0.001** |  | 1.00 (0.93, 1.09) | 0.906 |
| Q1 | Ref |  |  | Ref |  |
| Q2 | 1.30 (0.83, 2.04) | 0.256 |  | 0.83 (0.63, 1.08) | 0.161 |
| Q3 | 1.62 (1.05, 2.50) | **0.028** |  | 1.02 (0.80, 1.32) | 0.858 |
| Q4 | 1.47 (0.95, 2.29) | 0.084 |  | 1.03 (0.80, 1.32) | 0.812 |
| Q5 | 2.01 (1.32, 3.04) | **0.001** |  | 0.91 (0.70, 1.17) | 0.455 |
| P-trend |  | **0.001** |  |  | 0.967 |

HR was calculated by Cox model adjusted for age, age-squared, sex, ethnicity, TDI, smoking status, drinking status, education levels, physical activities, BMI, PRS, and total energy (if the exposure was energy or energy proportion, total energy intake will not be adjusted), C-reactive protein (CRP)

# Supplementary table 20. Sensitivity analyses for the association between UPF intake and risk of CD and UC when excluding participants with implausible energy intake or excluding participants with or incident CD or UC within the first year

|  | **Excluding participants with implausible energy intake** | |  | **Excluding participants with or incident CD or UC within the first year** | |
| --- | --- | --- | --- | --- | --- |
|  | **HR 95%CI** | **P** |  | **HR 95%CI** | **P** |
| CD |  |  |  |  |  |
| Serving |  |  |  |  |  |
| Per SD | 1.16 (1.03, 1.32) | **0.016** |  | 1.18 (1.04, 1.34) | **0.010** |
| Q1 | Ref |  |  | Ref |  |
| Q2 | 0.90 (0.57, 1.42) | 0.655 |  | 0.92 (0.58, 1.47) | 0.740 |
| Q3 | 1.06 (0.68, 1.66) | 0.785 |  | 1.14 (0.72, 1.80) | 0.571 |
| Q4 | 1.68 (1.10, 2.56) | **0.015** |  | 1.77 (1.15, 2.72) | **0.010** |
| Q5 | 1.58 (1.01, 2.46) | **0.045** |  | 1.63 (1.03, 2.57) | **0.037** |
| P-trend |  | **0.002** |  |  | **0.002** |
| Energy |  |  |  |  |  |
| Per SD | 1.18 (1.05, 1.32) | **0.006** |  | 1.15 (1.02, 1.30) | **0.021** |
| Q1 | Ref |  |  | Ref |  |
| Q2 | 0.97 (0.63, 1.49) | 0.903 |  | 1.03 (0.66, 1.59) | 0.902 |
| Q3 | 0.94 (0.61, 1.45) | 0.790 |  | 0.94 (0.60, 1.48) | 0.804 |
| Q4 | 1.39 (0.94, 2.07) | 0.102 |  | 1.49 (0.99, 2.23) | 0.055 |
| Q5 | 1.48 (1.00, 2.19) | 0.051 |  | 1.46 (0.97, 2.20) | 0.069 |
| P-trend |  | **0.010** |  |  | **0.014** |
| Energy% |  |  |  |  |  |
| Per SD | 1.24 (1.09, 1.40) | **0.001** |  | 1.25 (1.10, 1.41) | **0.001** |
| Q1 | Ref |  |  | Ref |  |
| Q2 | 1.28 (0.82, 2.01) | 0.277 |  | 1.35 (0.84, 2.15) | 0.211 |
| Q3 | 1.61 (1.05, 2.48) | **0.029** |  | 1.71 (1.10, 2.68) | **0.018** |
| Q4 | 1.43 (0.92, 2.22) | 0.116 |  | 1.51 (0.95, 2.38) | 0.079 |
| Q5 | 2.03 (1.34, 3.07) | **0.001** |  | 2.10 (1.36, 3.23) | **0.001** |
| P-trend |  | **0.001** |  |  | **0.001** |
| UC |  |  |  |  |  |
| Serving |  |  |  |  |  |
| Per SD | 0.99 (0.90, 1.08) | 0.810 |  | 0.97 (0.88, 1.06) | 0.510 |
| Q1 | Ref |  |  | Ref |  |
| Q2 | 0.99 (0.75, 1.31) | 0.926 |  | 0.96 (0.72, 1.28) | 0.785 |
| Q3 | 1.26 (0.96, 1.65) | 0.096 |  | 1.21 (0.92, 1.60) | 0.171 |
| Q4 | 1.00 (0.75, 1.34) | 0.981 |  | 0.98 (0.73, 1.31) | 0.873 |
| Q5 | 1.03 (0.77, 1.39) | 0.825 |  | 0.98 (0.72, 1.33) | 0.898 |
| P-trend |  | 0.862 |  |  | 0.900 |
| Energy |  |  |  |  |  |
| Per SD | 1.05 (0.97, 1.14) | 0.209 |  | 1.06 (0.98, 1.15) | 0.175 |
| Q1 | Ref |  |  | Ref |  |
| Q2 | 0.85 (0.65, 1.12) | 0.252 |  | 0.89 (0.68, 1.18) | 0.417 |
| Q3 | 1.01 (0.78, 1.30) | 0.958 |  | 1.03 (0.79, 1.35) | 0.827 |
| Q4 | 1.05 (0.82, 1.36) | 0.684 |  | 1.06 (0.81, 1.38) | 0.693 |
| Q5 | 1.07 (0.83, 1.38) | 0.613 |  | 1.09 (0.84, 1.42) | 0.511 |
| P-trend |  | 0.260 |  |  | 0.264 |
| Energy% |  |  |  |  |  |
| Per SD | 1.00 (0.93, 1.09) | 0.932 |  | 1.01 (0.93, 1.09) | 0.886 |
| Q1 | Ref |  |  | Ref |  |
| Q2 | 0.83 (0.64, 1.09) | 0.184 |  | 0.81 (0.61, 1.06) | 0.130 |
| Q3 | 1.02 (0.79, 1.31) | 0.892 |  | 1.02 (0.78, 1.32) | 0.896 |
| Q4 | 1.06 (0.82, 1.36) | 0.652 |  | 1.00 (0.77, 1.30) | 0.994 |
| Q5 | 0.90 (0.70, 1.17) | 0.446 |  | 0.91 (0.70, 1.18) | 0.476 |
| P-trend |  | 0.925 |  |  | 0.993 |

HR was calculated by Cox model adjusted for age, age-squared, sex, ethnicity, TDI, smoking status, drinking status, education levels, physical activities, BMI, PRS, and total energy (if the exposure was energy or energy proportion, total energy intake will not be adjusted)

# Supplementary table 21. Sensitivity analyses for the association between UPF intake and risk of CD and UC when using multiple imputation to process covariate

|  | **CD** | |  | **UC** | |
| --- | --- | --- | --- | --- | --- |
|  | **HR 95%CI** | **P** |  | **HR 95%CI** | **P** |
| Serving |  |  |  |  |  |
| Per SD | 1.16 (1.01, 1.33) | **0.034** |  | 0.97 (0.87, 1.07) | 0.502 |
| Q1 | Ref |  |  | Ref |  |
| Q2 | 0.99 (0.61, 1.61) | 0.959 |  | 1.00 (0.73, 1.36) | 0.992 |
| Q3 | 1.16 (0.72, 1.89) | 0.538 |  | 1.29 (0.96, 1.74) | 0.090 |
| Q4 | 1.84 (1.16, 2.91) | **0.010** |  | 0.95 (0.69, 1.31) | 0.745 |
| Q5 | 1.67 (1.03, 2.71) | **0.041** |  | 1.01 (0.73, 1.40) | 0.963 |
| P-trend |  | **0.003** |  |  | 0.871 |
| Energy |  |  |  |  |  |
| Per SD | 1.15 (1.02, 1.31) | **0.026** |  | 1.04 (0.96, 1.14) | 0.336 |
| Q1 | Ref |  |  | Ref |  |
| Q2 | 0.96 (0.60, 1.52) | 0.854 |  | 0.87 (0.65, 1.17) | 0.358 |
| Q3 | 0.98 (0.62, 1.55) | 0.931 |  | 0.95 (0.71, 1.27) | 0.739 |
| Q4 | 1.34 (0.87, 2.06) | 0.183 |  | 1.07 (0.81, 1.42) | 0.639 |
| Q5 | 1.46 (0.96, 2.23) | 0.080 |  | 1.04 (0.79, 1.38) | 0.776 |
| P-trend |  | **0.022** |  |  | 0.385 |
| Energy% |  |  |  |  |  |
| Per SD | 1.24 (1.09, 1.42) | **0.002** |  | 0.99 (0.90, 1.08) | 0.792 |
| Q1 | Ref |  |  | Ref |  |
| Q2 | 1.35 (0.84, 2.17) | 0.212 |  | 0.87 (0.65, 1.17) | 0.362 |
| Q3 | 1.53 (0.96, 2.43) | 0.075 |  | 1.01 (0.76, 1.33) | 0.944 |
| Q4 | 1.40 (0.87, 2.25) | 0.166 |  | 1.06 (0.80, 1.39) | 0.690 |
| Q5 | 1.98 (1.27, 3.09) | **0.003** |  | 0.85 (0.64, 1.14) | 0.274 |
| P-trend |  | **0.005** |  |  | 0.699 |

HR was calculated by Cox model adjusted for age, age-squared, sex, ethnicity, TDI, smoking status, drinking status, education levels, physical activities, BMI, PRS, and total energy (if the exposure was energy or energy proportion, total energy intake will not be adjusted)

# Supplementary table 22. Characteristics of summarized studies of associations between UPF intake and risk of IBD

| **Reference** | **Geography** | **Cohort size** | **Age** | **Sex**  **(% female)** | **Median FU**  **time** | **Incident IBD cases** | **Method**  **of dietary assessment** | **Intake of UPF** | **Effect on IBD incidence** | **Effect on IBD prevalent cases** |
| --- | --- | --- | --- | --- | --- | --- | --- | --- | --- | --- |
| Vasseur et al. 2021 | France | ﻿105,832 | Median 43.3 (+/-14.7) | 78% | 2.3 years | 75  (27 CD and 48 UC) | 24-hour dietary records | Proportion  of UPFs | ﻿HR for IBD risk: 1.44 (95% CI: 0.70 - 2.94, p=0.30) (for highest vs. lowest tertile) | Not reported |
| Nurala et al. 2021 | ﻿Argentina,Bangladesh,  Brazil, Canada, Chile, China, Colombia, India, Iran, Malaysia, Palestine,Pakistan, Philippines,Poland,  South Africa, Saudi Arabia, Sweden, Tanzania, Turkey, United Arab Emirates,  and Zimbabwe | ﻿116,087 | Range 35-70 years | 59.2% | 9.7 years | 467  (90 CD and 377 UC) | Food frequency questionnaires | Number of servings per day | ﻿HR for IBD risk: 1.92 (95% CI: 1.28 - 2.90, p=0.004) HR for CD risk: ﻿4.90 (95% CI: 1.78 - 13.4, p= 0.008) HR for UC risk: ﻿1.52 (95% CI: 0.96 - 2.41, p= 0.06)  (for ≥5 vs. <1 serving per day) | Not reported |
| Lo et al. 2021 | USA | 245.112 | ﻿Mean 56 years (range: 29–85) | 83.0% | Not reported | 857  (369 CD and 488 UC) | Food frequency questionnaires | Percentage of energy intake from UPFs | HR for CD risk: ﻿1.70 (95% CI:1.23 - 2.35, p= 0.0008) HR for UC risk: ﻿1.20 (95% CI: 0.91 - 1.58, p= 0.25)  (﻿for highest vs. lowest quartile) | Not reported |
| Chen and Wellens et al. | UK | 187.854 | Median 56.2 (SD 7.9) | 54.8% | 9.8 years | 841 (251 CD, 590 UC) | 24-hour dietary records | Number of servings, proportion of energy intake and proportion of energy percentage from UPFs | HR for CD risk (95%CI: 1.32 - 3.03, p=0.001) | OR for CD risk: 1.94 (95%CI: 1.52 - 2.49, p<0.001)  OR for UC risk: 1.39 (95%CI: 1.17 - 1.65, p<0.001) |

FU, follow-up; IBD, inflammatory bowel disease; UPF, ultraprocessed food; CD, Crohn’s disease; UC. Ulcerative colitis; HR, hazard ratio; OR, odds ratio; SD, standard deviation

**References**

1. Perez-Cornago A, Pollard Z, Young H, van Uden M, Andrews C, Piernas C, Key TJ, Mulligan A, Lentjes M. Description of the updated nutrition calculation of the Oxford WebQ questionnaire and comparison with the previous version among 207,144 participants in UK Biobank. Eur J Nutr. 2021 Oct;60(7):4019-4030. doi: 10.1007/s00394-021-02558-4. Epub 2021 May 6. PMID: 33956230; PMCID: PMC8437868.

2. Ministry of Agriculture, Fisheries and Food (1993) Food Portion Sizes, 2nd ed. London: HMSO.

3. Food Standards Agency (2002) McCance and Widdowson’s the Composition of Foods, 6th ed. Cambridge: Royal Society of Chemistry.
